# Integrating data-driven and physics-based approaches for robust wind power prediction: A comprehensive ML-PINN-Simulink framework

**DOI:** 10.1038/s41598-025-13306-7

**Published:** 2025-08-08

**Authors:** Rajaperumal T. A, Christopher Columbus Chinnappan

**Affiliations:** 1 School of Electrical Engineering, Vellore Institute of Technology, Chennai, Tamil Nadu India; 2 School of Computer Science and Engineering, Vellore Institute of Technology, Chennai, Tamil Nadu India

**Keywords:** Wind power forecasting, Machine learning, Physics-Informed neural network, Time series forecasting, Sustainable energy systems, Grid stability, Engineering, Energy science and technology, Renewable energy, Computational science

## Abstract

This study presents a comprehensive hybrid forecasting framework that synergizes machine learning algorithms, MATLAB Simulink-based physical modeling, and Physics-Informed Neural Networks (PINNs) to advance wind power prediction accuracy for a 10 kW Permanent Magnet Synchronous Generator (PMSG)-based Wind Energy Conversion System (WECS). Using a complete annual dataset of 8,760 hourly wind speed observations from the MERRA-2 platform, ten machine learning algorithms were systematically evaluated, including Random Forest, XGBoost, and an advanced Stacking ensemble model. The Stacking ensemble demonstrated superior performance, achieving an exceptional R^2^ of 0.998 and RMSE of 0.11, significantly outperforming individual algorithms. A detailed MATLAB Simulink model was developed to replicate turbine behaviour under identical wind conditions, physically, providing robust validation for ML predictions. The Simulink model achieved satisfactory performance under nominal wind conditions but exhibited computational constraints during extreme wind scenarios, leading to compromised output reliability. To bridge the gap between pure data-driven learning and physical realism, a Physics-Informed Neural Network was subsequently integrated to combine data-driven learning with physical constraints, using both observational data and physics-based synthetic datasets. Comparative analysis revealed that ML models deliver superior speed and accuracy for operational forecasting, while the PINN framework maintains physical consistency with competitive predictive performance. The framework’s practical applicability was demonstrated through a 2026 case study for southern Tamil Nadu, which incorporated projected environmental changes, including a 0.6% annual decline in wind speed. This real-world validation showcased the framework’s adaptability to evolving climatic conditions and long-term forecasting capabilities. This integrated methodology provides a robust foundation for enhancing wind power integration into modern energy systems, while maintaining both computational accuracy and physical interpretability, thereby supporting sustainable energy transition goals.

## Introduction

The growing need for clean and sustainable energy has established wind power as a cornerstone of the renewable energy sector^1^. As one of the fastest-growing renewable energy options, wind power plays a crucial role in reducing carbon emissions and enabling the transition to sustainable energy systems. However, the inherent variability and unpredictability of wind create significant challenges for maintaining power grid stability and effectively managing energy resources^2,3^. Wind power fluctuations can cause frequency deviations and voltage instability, making accurate forecasting essential for grid operators to anticipate variations and maintain system balance. Precise forecasting enables efficient grid management, resource planning, improved energy dispatch, and risk minimization through optimized scheduling and generation pattern prediction. Beyond grid stability, wind forecasting is vital for smart grid applications, microgrid integration, real-time energy trading, market clearing mechanisms, and hybrid renewable systems with storage components. From an economic perspective, accurate forecasting reduces operational costs for utilities, enhances revenue for wind farm operators, and facilitates efficient energy trading by minimizing dependence on costly spinning reserves.

Wind Energy Conversion Systems (WECS), particularly those utilizing Permanent Magnet Synchronous Generators (PMSG), are widely adopted due to their efficiency, reliability, and adaptability to fluctuating wind conditions^4^. Accurately predicting the power output of these systems requires a comprehensive understanding of wind speed trends and their complex interactions with turbine dynamics. This complexity has driven active research in wind power forecasting, which integrates statistical, physical, and machine learning approaches to address the nonlinear and dynamic characteristics of wind behavior^3^. Traditional forecasting methods, including persistence models and numerical weather predictions, often fail to capture these complexities, necessitating the use of advanced machine learning and simulation-based approaches to address them. Various methodologies exist across the spectrum: statistical models such as ARIMA, physical models based on meteorological data, and machine learning techniques, each offering different levels of accuracy and computational requirements. Machine learning algorithms have proven particularly effective in analyzing extensive datasets of wind speeds and identifying underlying patterns that affect energy production. Ensemble methods like XGBoost and Random Forest effectively handle non-linear relationships and data variability^5,6^, while deep learning approaches such as LSTM and hybrid CNN-LSTM networks excel at capturing temporal dependencies, though they require substantial computational resources and large datasets^6^. To enhance and validate machine learning forecasts, this study integrates MATLAB Simulink simulation as a robust framework for validation. The MATLAB Simulink platform provides comprehensive modeling capabilities for analyzing the physical and electrical dynamics of WECS under realistic conditions. The simulation model encompasses wind turbine aerodynamics, which transform wind velocity into mechanical energy, PMSG dynamics that convert mechanical energy into electrical power, and the complete energy conversion process. This integration bridges the gap between traditional simulations that rely on fixed physical models and ML approaches that learn from real-time weather and historical data, enabling more precise forecasting under rapidly changing environmental conditions^7^.

The key contributions of this research include utilizing real-time wind data from the MERRA-2 open-access platform for model training, implementing and comparing multiple ML algorithms, and validating ML forecasts against detailed MATLAB simulation models. Performance evaluation employs indicators such as Mean Absolute Error (MAE), Root Mean Square Error (RMSE), and Mean Absolute Percentage Error (MAPE) to assess alignment between machine learning predictions, simulation results, and actual data^8,9^. The study also explores emerging techniques such as physics-informed neural networks (PINNs), which integrate physical laws into neural network training to create hybrid learning models that improve forecast interpretability and robustness^10^. Accurate wind energy forecasting supports multiple Sustainable Development Goals, including SDG 7 (Affordable and Clean Energy) by enhancing the reliability of renewable energy, SDG 13 (Climate Action) by reducing carbon emissions, and SDG 9 (Industry, Innovation, and Infrastructure) by promoting technological advancements. Ultimately, these advancements in wind energy forecasting are critical for ensuring a stable, efficient, and economically viable transition toward a renewable energy future.

## Literature survey

The growing focus on sustainable and clean energy sources has made wind energy a key technology in the field of renewable energy. A key factor in reducing greenhouse gas emissions and facilitating the transition to a sustainable energy environment is wind power, one of the renewable energy sources with the fastest growth rate. However, power grid stability and energy resource management are severely hampered by wind’s intrinsic unpredictability and fluctuation. Overcoming these obstacles, guaranteeing dependable grid operations, improving resource allocation, and cutting operating expenses all depend on accurate wind power forecasts. Permanent Magnet Synchronous Generators (PMSGs) are utilized in Wind Energy Conversion Systems (WECS), which are gaining popularity due to their high efficiency, dependability, and flexibility in responding to changing wind conditions. Due to the PMSG’s design, there is no need for a gearbox, which reduces maintenance requirements and mechanical losses^11,12^. The non-linear connection between wind velocity and power production in PMSG-based WECS performance is heavily reliant on precise wind speed predictions^13,14^. Conventional statistical forecasting methods, such as persistence models and autoregressive integrated moving average (ARIMA) models, frequently fail to capture the intricate dynamics of wind speed variability^15,16^.

### Machine learning in wind power forecasting

Machine learning (ML) has emerged as a powerful tool for wind power forecasting, leveraging historical wind speed records to identify complex patterns that influence energy production^17,18^. Various ML algorithms, such as Adaptive Boosting (AdaBoost), are an ensemble learning method that enhances weak learners by adjusting the weights of misclassified instances to improve prediction accuracy^18^. A decision tree is a supervised learning algorithm that splits data based on feature values, providing an interpretable structure,however, it can be prone to overfitting without proper tuning^19^. Extremely Randomized Trees (Extra Trees) is an ensemble method that introduces additional randomness into the decision-making process, which helps reduce overfitting and improve model generalization^20^. Gradient Boosting builds models sequentially, with each model correcting the errors of the previous one, making it effective for capturing complex relationships in the data^17^. K-Nearest Neighbours (KNN) classifies data based on the majority class of its nearest neighbours, though it can be computationally expensive for large datasets^21^.

Linear Regression (LR) models the relationship between variables through a linear equation and is often used as a baseline for regression tasks, but it may not capture more complex relationships^22^. Neural Networks (NN), inspired by the human brain, consist of layers of interconnected neurons that can learn complex patterns, but they require large datasets and significant computational resources for training^23,24^. Random Forest and XGBoost, excel in handling high data variability and capturing non-linear relationships, making them effective in predicting fluctuating wind power outputs^19,23^. However, achieving high-accuracy wind power forecasts requires overcoming several constraints. ML models demand extensive training data, which may not always be readily available or of sufficient quality. Moreover, tuning hyperparameters, selecting appropriate features, and designing network architectures significantly impact the model’s predictive performance^21,22^.

Another critical aspect is uncertainty quantification, as wind speed and power generation are inherently stochastic. Uncertainty estimation techniques, such as probabilistic forecasting and ensemble methods, help in assessing model reliability and improving decision-making in energy management systems. Despite these advancements, several barriers hinder the widespread adoption of ML-based forecasting models^25^. In summary, while ML-driven wind power forecasting offers significant improvements over traditional methods, its effectiveness depends on overcoming data constraints, improving uncertainty quantification, addressing computational limitations, and enhancing model interpretability for practical applications in the renewable energy sector.

### Simulation-based modelling with MATLAB simulink

Beyond machine learning-based predictions, simulation frameworks, particularly MATLAB Simulink, provide critical insights into the electrical and WECS systems. These platforms enable comprehensive analysis of system behavior through high-fidelity dynamic modeling that captures the complex interactions between aerodynamic, mechanical, and electrical components^26,27^. The foundation of effective WECS simulation lies in accurate aerodynamic modeling, where MATLAB Simulink excels by incorporating essential characteristics that govern the transformation of wind energy into mechanical power. The platform’s capability to model aerodynamic parameters, such as the power coefficient (Cp), tip-speed ratio (λ), and blade pitch angle, provides a crucial understanding of energy capture efficiency across varying operational conditions^28,29^. Recent advancements in Simulink toolboxes, specifically designed for renewable energy systems, have further enhanced these capabilities, particularly through real-time hardware-in-the-loop (HIL) simulation features that bridge the gap between theoretical modeling and practical implementation^30^.

The integration of PMSG dynamics within these simulation frameworks creates a comprehensive representation of the entire energy conversion process. This integration is particularly valuable, as it enables the validation of machine learning predictions against real-world operating conditions while supporting the development of supervisory control. PMSG models typically employ d-q axis modeling in the synchronous reference frame and incorporate variable-speed control strategies, making them well-suited for grid integration studies and providing insights into system behavior under diverse operational scenarios^31,32^. However, achieving simulation accuracy requires careful attention to several modeling constraints and considerations. The aerodynamic modeling component must accurately capture the complex fluid dynamics governing wind-turbine interactions, where recent developments in blade element momentum theory (BEM) and turbulence modeling have significantly enhanced the realism of simulations^33,34^. Similarly, accurate PMSG modeling demands consideration of electromechanical energy conversion behavior, efficiency variations across operating ranges, and operational reliability under varying wind and load conditions. Advanced implementations sometimes incorporate thermal effects and magnetic saturation to enhance the realism of generator behavior further^35,36^.

The challenge of parameter uncertainty poses a significant concern for the accuracy of WECS simulations. Fluctuating wind profiles, varying air density, and system losses introduce uncertainties that can substantially impact simulation results. Addressing these uncertainties requires robust parameter estimation techniques, comprehensive sensitivity analyses, and thorough validation against experimental data or supervisory control and data acquisition (SCADA) field measurements^7^. The increasing adoption of digital twin implementations and co-simulation approaches with machine learning models reflects the industry’s response to these accuracy challenges, offering more sophisticated methods for handling parameter uncertainties and model validation. Despite these advances, MATLAB Simulink-based WECS modeling faces several persistent challenges that impact practical implementation. Computational complexity becomes a significant constraint when incorporating multi-domain, high-fidelity models that span the entire turbine-drivetrain-generator-grid interface, often resulting in prohibitively long simulation times. Researchers have addressed this issue through various strategies, including model order reduction techniques and parallel simulation approaches; however, these solutions often involve trade-offs between computational efficiency and model fidelity^37^.

Data availability and quality represent equally critical challenges in achieving accurate simulations. While the integration of real-time wind data from meteorological datasets, such as MERRA-2, or on-site SCADA records can significantly enhance simulation realism, these data sources require extensive preprocessing and careful synchronization with the simulation timelines. The presence of inadequate or noisy data can lead to parameter drift, which fundamentally undermines prediction reliability and creates a cascade of errors throughout the simulation process. The necessity for model simplifications to manage computational burden creates an inherent tension in WECS simulation design. While simplifications are essential for practical implementation, they can significantly limit the granularity of transient behavior prediction, potentially missing critical system dynamics that occur during rapid wind changes or grid disturbances. This limitation has driven the development of hybrid simulation-machine learning approaches that leverage the strengths of both methodologies while mitigating their weaknesses.

Contemporary research increasingly focuses on addressing these challenges through integrated approaches that combine optimization algorithms for parameter tuning with advanced control strategies. The emergence of reinforcement learning applications in WECS control represents an auspicious direction, offering adaptive solutions that can learn and optimize system behavior in real-time. These developments suggest that future WECS modeling will likely rely on sophisticated hybrid frameworks that seamlessly integrate high-fidelity physics-based simulation with data-driven machine learning approaches, creating more robust and accurate tools for wind energy system analysis and optimization. Despite these ongoing challenges, MATLAB Simulink maintains its position as a powerful and adaptable tool for comprehensive WECS analysis, continuing to provide detailed insights into the complex interactions between turbine aerodynamics, generator dynamics, and energy conversion efficiency. The platform’s versatility in handling multi-domain modeling, combined with its extensive library of specialized toolboxes, makes it an invaluable resource for researchers and engineers working to advance wind energy technology. However, the path forward requires systematic approaches to overcome fundamental modeling constraints, develop more sophisticated methods for mitigating parameter uncertainties, and implement innovative strategies for managing computational loads—all essential elements for improving the reliability of wind power forecasting and enhancing overall system performance^38^.

The evolution of WECS modeling appears to be heading toward increasingly sophisticated hybrid approaches that leverage the complementary strengths of physics-based simulation and data-driven methodologies. Among the most promising developments in this direction is the emerging application of physics-informed neural networks (PINNs), which offer the potential to create unified modeling frameworks that couple high-fidelity simulation data with machine learning predictions. This approach promises to deliver both the interpretability of traditional physics-based models and the adaptability of modern machine learning techniques, potentially revolutionizing how wind energy systems are modeled, analyzed, and optimized^10^. Such integrated approaches represent a natural evolution in the field, addressing the limitations of purely simulation-based or purely data-driven methods while opening new possibilities for more accurate, efficient, and comprehensive wind energy system analysis.

### Physics-informed neural networks (PINN) in wind power forecasting

PINNs signify a transformative advancement in scientific machine learning, integrating the adaptability of neural networks with the precision of physical laws. They uniquely address supervised learning tasks while adhering to the principles of physics as articulated by general nonlinear partial differential equations^39^. As universal function approximators, these networks incorporate the knowledge of physical laws governing a dataset into the learning process, rendering them particularly advantageous for engineering applications where adherence to physical constraints is critical. This embedded physics knowledge serves as a regularization mechanism during neural network training, effectively mitigating overfitting and enhancing generalization capabilities, particularly in sparse data scenarios prevalent in engineering contexts^40^.

Since their inception in 2019 by Raissi et al., physics-informed neural networks have become pivotal in scientific machine learning, facilitating the efficient resolution of ordinary and partial differential equations using sparse measurements, while experiencing rapid advancements in training methodologies and architectural innovations. In the realm of renewable energy forecasting, particularly wind power prediction, PINNs offer distinct advantages by addressing the inherent limitations of purely data-driven approaches through the integration of domain-specific physical knowledge. This feature is particularly beneficial for wind energy applications where high-quality training data may be scarce or costly to acquire, as the incorporation of physical laws compensates for data scarcity while maintaining predictive accuracy. The benefits of PINNs for wind energy forecasting applications are multifaceted and interrelated. Enhanced interpretability is a primary advantage, as PINNs ensure that predictions align with established physical laws, which is essential for fostering trust in forecasting systems employed in critical grid operations^41^.

This interpretability naturally extends to improved robustness in scenarios with limited training data or extrapolation, addressing common challenges in renewable energy applications where comprehensive historical data may not be available for all operating conditions. Moreover, PINNs exhibit exceptional proficiency in managing the multi-physics problems inherent in wind energy systems, effectively modeling the intricate coupling between meteorological conditions, turbine aerodynamics, and electrical power generation through a holistic approach that considers the interdependencies among various physical phenomena^42^. This comprehensive modeling capability facilitates more accurate predictions by capturing the full spectrum of physical interactions that govern wind energy conversion processes, ultimately leading to more reliable and physically consistent forecasting outcomes.

### Contribution of the proposed work

Recent advancements in wind power forecasting have been significantly enhanced through the application of ML models, MATLAB Simulink-based simulations, and PINNs. ML algorithms, including Random Forest and XGBoost, have demonstrated high accuracy in capturing the nonlinear and stochastic characteristics of wind speed and power generation. Simultaneously, MATLAB Simulink facilitates detailed modeling of the electromechanical and aerodynamic dynamics of WECS, particularly those employing PMSGs. PINNs are effective in modeling wind energy systems by addressing multi-physics problems, integrating meteorological conditions, turbine aerodynamics, and power generation through their interdependent relationships. Despite their strengths, these methodologies are predominantly explored in isolation. This separation limits the validation of ML predictions under realistic operational scenarios and restricts comprehensive assessments of forecasting accuracy, computational efficiency, and scalability. Moreover, there is a scarcity of comparative studies utilizing a standard dataset to evaluate both data-driven and physics-based models under identical wind conditions. Consequently, a critical research gap exists in the absence of an integrated hybrid framework that combines ML-based forecasting with Simulink simulation. Addressing this gap would facilitate robust cross-validation, enhance the reliability of wind power prediction, and contribute to the development of scalable and sustainable energy management systems.

This study introduces a novel hybrid forecasting framework that systematically integrates machine learning models with MATLAB Simulink-based physical simulations to significantly enhance both the accuracy and computational efficiency of wind power predictions. The framework represents a paradigm shift from traditional single-approach methodologies by establishing a comprehensive three-stage architecture that strategically leverages the complementary strengths of data-driven learning and physics-based modeling. The research makes several distinct contributions to the field of wind energy forecasting.

In Case 1, it provides a rigorous comparative analysis of ten different machine learning algorithms using real-time wind data from the MERRA-2 dataset, encompassing 8,760 hourly observations. This comprehensive evaluation identifies Random Forest, XGBoost, and Stacking ensemble models as the most effective approaches, with each achieving exceptional predictive accuracy characterized by R^2^ values exceeding 0.99 and demonstrating remarkably low mean absolute error (MAE) and root mean square error (RMSE) metrics. This analysis definitively establishes the superiority of ensemble learning methods in capturing the complex, nonlinear, and variable characteristics inherent in wind power generation.

In Case 2, the study develops a sophisticated MATLAB Simulink model of a 10 kW PMSG-based wind energy conversion system that integrates critical subsystems, including aerodynamic, drivetrain, generator, and control components. This comprehensive physical model enables the accurate simulation of wind energy conversion dynamics, providing a robust platform for direct comparison of performance with machine learning approaches. The research demonstrates that while physical simulations maintain strong consistency with engineering principles, they face significant computational limitations and scalability challenges, particularly when processing extended datasets or operating under dynamic high-wind conditions.

In Case 3, the framework introduces an innovative application of PINNs that seamlessly integrates physical laws into neural network architectures, ensuring both accuracy and generalizability even with limited training data. The PINN implementation utilizes a hybrid data approach, combining real-time MERRA-2 wind data with synthetic data generated from the physical model, creating a comprehensive dataset that encompasses critical variables such as mechanical torque, electrical torque, pitch angle, rotor speed, and angular velocity. This physics-informed approach enhances model reliability by maintaining consistency with fundamental engineering principles throughout the prediction process while demonstrating superior computational efficiency and faster convergence compared to traditional neural networks.

In Case 4, the research further advances the field by implementing a forward-looking forecasting capability that incorporates real-world environmental factors. Specifically, the study integrates findings from the Council on Energy, Environment, and Water (CEEW), indicating an annual reduction of up to 0.6% in average wind speeds across southern Tamil Nadu. This decline factor is applied to generate realistic 2026 wind speed forecasts. These predicted wind speeds serve as input parameters for both the MATLAB Simulink-based physical model and the PINN model, allowing for comprehensive simulations of electricity generation across various modeling approaches. The framework then conducts a systematic performance evaluation by comparing the electricity generation forecasts produced by the machine learning models, the physics-based Simulink simulation, and the PINN approach, thereby providing a comprehensive assessment of the accuracy and reliability of each methodology in practical wind-based electricity generation forecasting scenarios. This multi-model comparison approach demonstrates practical applicability by addressing long-term environmental trends that significantly impact wind energy planning and deployment strategies while establishing benchmark performance metrics across different modeling paradigms.

Perhaps most significantly, this work establishes a unified methodology that bridges the traditional divide between physical and data-driven modeling approaches. The proposed framework delivers a scalable, accurate, and computationally efficient solution that addresses the critical need for real-time wind power forecasting in operational environments. By demonstrating how machine learning models can provide superior forecasting accuracy while maintaining computational efficiency, and how physics-informed approaches can enhance reliability and interpretability, the research provides a comprehensive roadmap for next-generation wind energy forecasting systems.

The broader impact of this research extends beyond technical contributions to support global sustainability initiatives. The framework directly advances the development of intelligent renewable energy systems that align with international sustainability goals, particularly the United Nations Sustainable Development Goals 7 (Affordable and Clean Energy) and 13 (Climate Action). By providing more accurate and efficient wind power forecasting capabilities, this work contributes to the optimization of renewable energy integration, supporting the transition toward sustainable energy systems and enhanced grid stability.

This research establishes a new standard for hybrid modeling approaches in renewable energy forecasting, demonstrating that the integration of machine learning, physical simulation, and physics-informed neural networks can overcome the limitations of individual methodologies while maximizing their collective strengths. The framework’s proven scalability and real-time capability position it as a valuable tool for wind farm operators, energy system planners, and researchers working to advance the efficiency and reliability of wind energy systems globally.

## Methodology

This section provides a comprehensive examination of the methodologies employed in forecasting wind energy generation using the most effective machine learning models, such as Random Forest and XGBoost. The discussion encompasses data preprocessing, feature selection, model training, validation, and performance evaluation. Additionally, it addresses the modeling of various components of the WECS within MATLAB Simulink, including wind turbine dynamics, power electronics, and control strategies, to facilitate a thorough system analysis.

### Machine learning framework

**Fig. 1:**
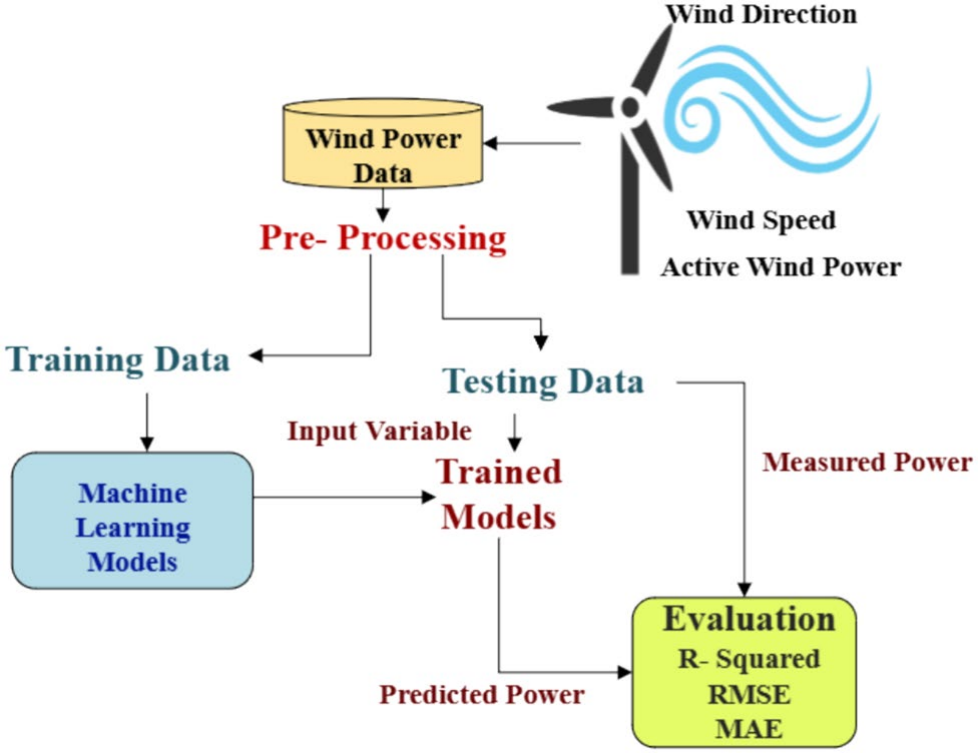
Workflow diagram for machine learning models.

Figure 1 illustrates a framework for predicting wind power utilizing machine learning models. Initially, wind power data undergoes pre-processing and is subsequently divided into training and testing datasets. The Random Forest and XGBoost models are developed using the training data. These trained models are then employed to forecast power output by evaluating the input variables from the testing dataset. The performance of the models is assessed by comparing the predicted and actual power outputs, using metrics such as R-squared, Root Mean Square Error (RMSE), and Mean Absolute Error (MAE).

The wind speed data from the MERRA-2 dataset constitutes the primary input feature for the ML models. An exploratory data analysis was performed to identify patterns and relationships within the data, and missing values were addressed to maintain data integrity. To enhance prediction accuracy, additional derived features, such as rolling averages and wind speed fluctuations, were engineered^43^. Preprocessing is a critical step in ensuring that raw wind power data is clean, consistent, and suitable for training machine learning models, involving several intricate processes. Missing values, which may arise from sensor failures, connectivity issues, or other data collection problems, were addressed using various techniques. This included mean or median imputation, which replaces missing values with the average or median of the corresponding variable, and interpolation methods such as linear or polynomial interpolation, which estimate missing values based on surrounding data patterns. Advanced machine learning models were also employed to predict missing values using features from other datasets. In instances where imputation was impractical, rows or attributes with a high percentage of missing data were removed.

Outlier detection and removal were performed to mitigate the impact of data points that significantly deviated from typical values, as such anomalies can distort model training. Techniques such as the Z-score method, interquartile range (IQR), and machine learning-based anomaly detection were employed. Since wind speed is measured in meters per second and often appears alongside variables with differing scales, normalization (scaling values between 0 and 1) or standardization (scaling to a mean of 0 and standard deviation of 1) was used to ensure that all features contributed equally during model training. Feature engineering was applied to enhance model performance by creating new variables that more accurately capture the underlying relationships in the data. For instance, wind direction and speed were combined to create vector representations, shifting medians and rolling averages were calculated to track temporal trends, and wind speeds were categorized into three classes: low, medium, and high. To ensure that only the most relevant variables were included, feature selection and dimensionality reduction techniques such as Principal Component Analysis (PCA), mutual information, and correlation analysis were utilized. In situations where data imbalance was detected (e.g., an abundance of instances of specific wind conditions), resampling strategies were implemented. Oversampling involved duplicating samples from the minority class, while under-sampling reduced the number of samples from the majority class to balance the dataset. Categorical variables such as wind turbine types were converted into numerical representations using label encoding or one-hot encoding.

For time-series data, maintaining temporal consistency was essential. This required proper alignment across time intervals and the use of consistent timestamps to preserve temporal integrity. Each of these preprocessing steps contributed to preparing high-quality, well-structured input data, enabling machine learning models to effectively identify patterns and improve forecasting performance^22^. Hyperparameter tuning played a pivotal role in optimizing the performance of the machine learning models. These hyperparameters, which are not learned during model training, govern model complexity and the learning process. Examples include the number of neighbours in K-Nearest Neighbours and the depth of decision trees in ensemble methods. In this study, hyperparameter optimization was performed using a combination of grid search and manual tuning based on cross-validation results. This process significantly improved model performance, particularly for ensemble models such as Random Forest and XGBoost. Careful adjustment of parameters such as n_estimators, max_depth, and learning_rate enabled these models to capture complex, non-linear patterns in wind energy data without overfitting. XGBoost achieved the best performance, with a testing Mean Absolute Error (MAE) of 0.035 and an R^2^ of 0.997. The optimized Random Forest model obtained a testing MAE of 0.027 and an R^2^ of 0.995. These results underscore the importance of fine-tuning key hyperparameters to achieve a balance between generalization and model complexity, particularly in dynamic renewable energy forecasting applications.

**Fig. 2:**
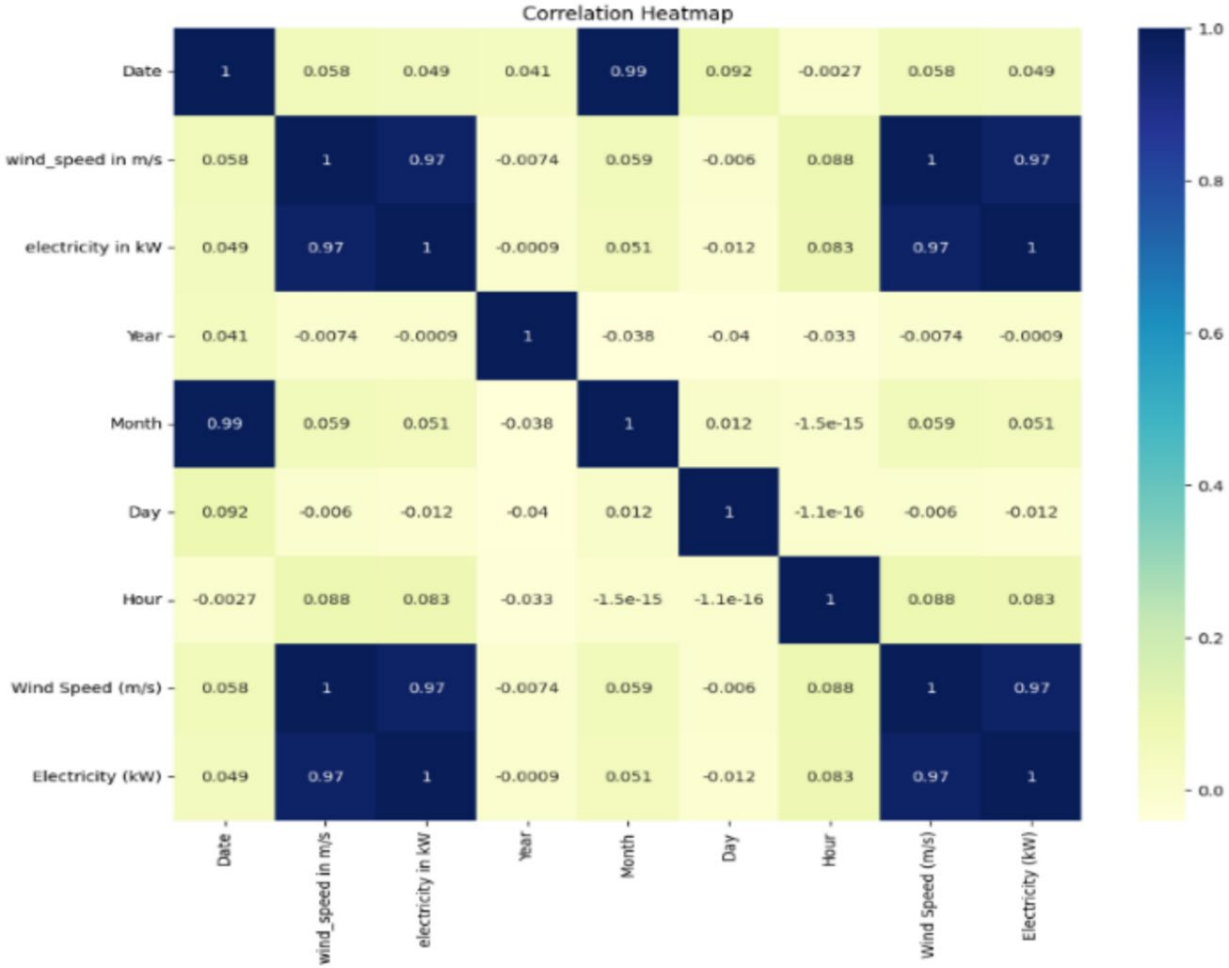
Correlation analysis of the attributes in the wind data set.

Figure 2 illustrates a correlation heatmap that highlights the relationships between various variables. Notably, there is a strong positive correlation between wind speed and electricity generation (r ≈ 0.97), indicating that increased wind speeds are associated with higher electricity production. Conversely, temporal variables such as month and date exhibit weaker correlations with wind speed and power output, suggesting that seasonal factors exert a limited influence on energy generation. The Comprehensive Preprocessing Summary of the dataset is:

- Initial dataset: 8,760 rows × 4 columns
- Final dataset: 8,694 rows × 23 columns
- Rows removed: 66
- Columns added: 17
- Missing values imputed: 0
- Outliers detected: 66
- Outliers removed: 66
- **Processing steps performed:**
- Added 17 temporal and cyclical features
- Detected 66 outliers, removed 66
- **Data transformation metrics:**
- Row change: −0.75%
- Column change: + 475.00%
- Data completeness: 100.00%

Table 1 presents a summary of the optimized hyperparameters for various machine learning models and a PINN. Each ML model is meticulously fine-tuned using fivefold cross-validation to ensure robust generalization and to mitigate overfitting to specific temporal or stochastic variations in the wind data. This cross-validation strategy facilitates the evaluation of models on multiple subsets of the dataset, thereby enhancing the reliability of performance estimates. Ensemble models, such as Random Forest, Gradient Boosting, and XGBoost, are configured with multiple estimators and regularization parameters. The tuning process for these models emphasizes capturing complex, non-linear interactions between wind features while maintaining generalization across validation folds. Simpler models, such as Linear Regression, serve as benchmarks and are evaluated using the same cross-validation protocol for consistency. The K-Nearest Neighbours model is tuned with distance-based weighting and validated across folds to more accurately model localized patterns in wind behaviour. The Neural Network architecture, comprising multiple hidden layers and employing adaptive optimization via the Adam solver, is trained and validated using Stratified K-Fold cross-validation to address non-uniform data distribution and potential class imbalance.

**Table 1:** Hyperparameters of Machine Learning Models.

Model	Tuned Parameters	Search Space
AdaBoost	n_estimators = 100, learning_rate = 0.1	n_estimators: [50, 100, 150, 200], learning_rate: [0.01, 0.05, 0.1, 0.5, 1.0]
Decision Tree	max_depth = 10, min_samples_split = 5, min_samples_leaf = 2	max_depth: [5, 10, 15, None], min_samples_split: [2, 5, 10], min_samples_leaf: [1, 2, 4]
Extra Trees	n_estimators = 150, max_depth = 12, min_samples_split = 4	n_estimators: [100, 150, 200], max_depth: [10, 12, 15], min_samples_split: [2, 4, 6]
Gradient Boosting	n_estimators = 200, learning_rate = 0.05, max_depth = 5	n_estimators: [100, 200, 300], learning_rate: [0.01, 0.05, 0.1], max_depth: [3, 5, 7]
K-Nearest Neighbors	n_neighbors = 5, weights = ’distance’, metric = ’minkowski’	n_neighbors: [3, 5, 7, 9], weights: [‘uniform’, ‘distance’], metric: [‘euclidean’, ‘manhattan’, ‘minkowski’]
Linear Regression	fit_intercept = True, normalize = False	fit_intercept: [True, False], normalize: [True, False]
Neural Network	hidden_layer_sizes = (100, 50), activation = ’relu’, solver = ’adam’, alpha = 0.0001	hidden_layer_sizes: [(50,), (100,), (100, 50)], activation: [‘relu’, ‘tanh’, ‘logistic’], solver: [‘adam’, ‘sgd’], alpha: [0.0001, 0.001]
Random Forest	n_estimators = 200, max_depth = 15, min_samples_split = 4, min_samples_leaf = 2	n_estimators: [100, 200, 300], max_depth: [10, 15, 20, None], min_samples_split: [2, 4, 6], min_samples_leaf: [1, 2, 4]
XGBoost	n_estimators = 300, learning_rate = 0.05, max_depth = 6, subsample = 0.8, colsample_bytree = 0.8	n_estimators: [100, 200, 300, 400], learning_rate: [0.01, 0.05, 0.1], max_depth: [4, 6, 8], subsample: [0.6, 0.8, 1.0], colsample_bytree: [0.6, 0.8, 1.0]
PINN	n_layers = 4, Hidden_unit_0 = 102, Hidden_unit_1 = 127, Hidden_unit_2 = 37, Hidden_unit_3 = 127, learning_rate = 0.00595, Physics_weight = 0.0243	n_layers = [2, 3, 4, 5, 6], Hidden_unit_0 [32, 64, 96, 128, 160], Hidden_unit_1 = [32, 64, 96, 128, 160], Hidden_unit_3 = [16, 32, 48, 64], learning_rate = [1e-4, 5e-4, 1e-3, 5e-3, 1e-2] (log-uniform sampling), [1e-3, 1e-2, 5e-2, 0.1, 0.2] (log-uniform sampling)

For the Physics-Informed Neural Network (PINN), cross-validation is adapted through a domain-specific split of the dataset into:

- A data subdomain for supervised loss,
- A physics-informed subdomain composed of collocation points enforcing PDE residuals, and

A validation subset is used to tune key hyperparameters such as the number of layers, neurons per layer, learning rate, and physics-constrained weighting. This structured validation ensures the PINN not only fits observational data but also adheres to the underlying physical laws governing wind energy generation. Collectively, the incorporation of cross-validation across all models strengthens the predictive rigor and ensures the hyperparameter choices yield models that generalize well to unseen wind energy data scenarios.

#### Data splitting

To ensure that the training set contains enough information for the model to learn patterns and that the testing set is hidden during training, the pre-processed data is randomly divided into 80% training and 20% testing subsets. This allows for an accurate assessment of the model’s generalization and predictive performance.ance^44^.

**Fig. 3:**
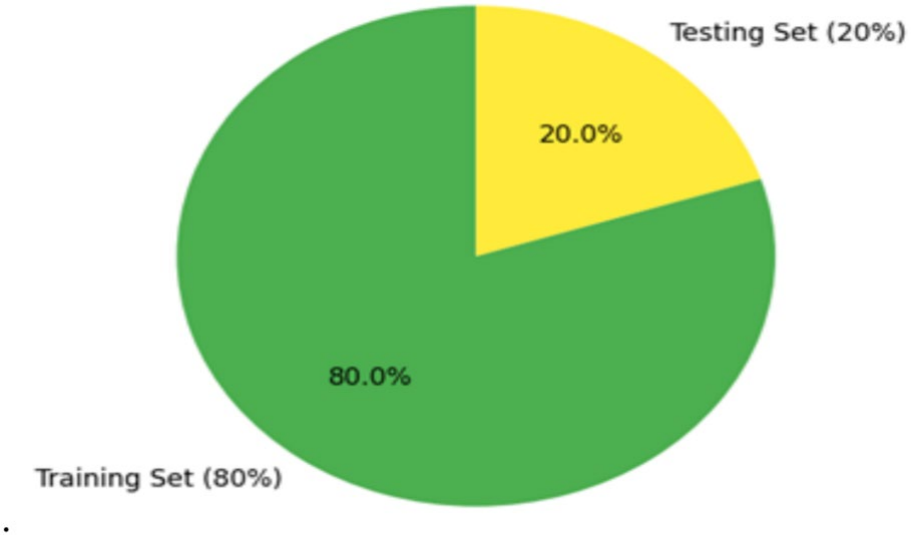
Data distribution diagram.

Figure 3 illustrates the data distribution used in the model development process. As shown, 80% of the dataset (in green) is allocated for training, while the remaining 20% (in yellow) is reserved for testing. This commonly adopted 80/20 split ensures that the model is trained on a substantial portion of the data while preserving a representative set for reliable performance evaluation and validation.

#### Evaluation

The trained models use input variables from the testing dataset to predict wind power output (Predicted Power)^44^. The actual power measured from the testing dataset is compared to the expected power outputs. The evaluation metrics are,

**Mean absolute error (MAE):**

Calculates the average size of forecast mistakes without taking direction into account.

1 $${\varvec{M}}{\varvec{A}}{\varvec{E}}=\frac{1}{{\varvec{n}}}\sum_{{\varvec{i}}=1}^{{\varvec{n}}}|{{\varvec{y}}}_{{\varvec{i}}}-{{\varvec{y}}}_{{\varvec{k}}}|$$

where, ${y}_{i}$ is the actual value, ${y}_{k}$ is the predicted value, and n is the number of samples.

**Mean Squared Error (MSE)**:

Increases the penalty for greater mistakes by squaring them.

2 $${\varvec{M}}{\varvec{S}}{\varvec{E}}=\frac{1}{{\varvec{n}}}\sum_{{\varvec{i}}=1}^{{\varvec{n}}}{({{\varvec{y}}}_{{\varvec{i}}}-{{\varvec{y}}}_{{\varvec{k}}})}^{2}$$

Root Mean Squared Error (RMSE):

Stands for the average squared difference between the expected and actual numbers, squared as a root.

3 $${\varvec{R}}{\varvec{M}}{\varvec{S}}{\varvec{E}}=\sqrt{\frac{1}{{\varvec{n}}}\sum_{{\varvec{i}}=1}^{{\varvec{n}}}{({{\varvec{y}}}_{{\varvec{i}}}-{{\varvec{y}}}_{{\varvec{k}}})}^{2}}$$

R-Squared (Coefficient of Determination):

Shows how well the model accounts for the variation in the data.

4 $${{\varvec{R}}}^{2}=1-\frac{\frac{1}{{\varvec{n}}}\sum_{{\varvec{i}}=1}^{{\varvec{n}}}{\left({{\varvec{y}}}_{{\varvec{i}}}-{{\varvec{y}}}_{{\varvec{k}}}\right)}^{2}}{\frac{1}{{\varvec{n}}}\sum_{{\varvec{i}}=1}^{{\varvec{n}}}{\left({{\varvec{y}}}_{{\varvec{i}}}-{{\varvec{y}}}^{|}\right)}^{2}}$$

where, ${y}^{|}$ is the mean of the actual values.

In order to provide accurate wind power forecasts, this iterative process makes sure the selected model is optimized, opening the door for ongoing developments in renewable energy forecasting and optimization^43^.

#### Mathematical modelling of stacking ensemble (RF + XGB)

Let,

X= [X_1_, X_2_,……,X_n_] ∈ ℝ^n×d^ be the input feature matrix.

y = [y_1_, y_2_,…….,y_n_] ∈ ℝ^n^ be the target values.

f_RF_ is the function learned by the Random Forest model.

f_XGB_ is the function learned by the Xtreme Gradient Boosting model.

f_meta_ is the meta-learner, trained on the outputs of RF and XGB.

**Base learners Predictions:**

For each sample X_i_:

5 $$y_{i}^{{\widehat{{}}(1)}} = f_{RF} (X_{i} )$$

6 $$y_{i}^{{\widehat{{}}(2)}} = f_{RF} (X_{i} )$$

**Meta-Feature Construction:**

7 $$Z_{i} = [y_{i}^{{\widehat{{}}(1)}} ,y_{i}^{{\widehat{{}}(2)}} ]$$

Let Z = [Z_1_,….., Z_n_] ∈ ℝ^n×2^

**Train Meta-Learner:**

The meta-learner is trained on (Z, y):

8 $$y_{i}^{{\widehat{{}}}} = f_{meta} (Z_{i} ) = f_{meta} ([y_{i}^{{\widehat{{}}(1)}} ,y_{i}^{{\widehat{{}}(2)}} ])$$

**f**_**meta**_ is the Ridge Regression model

### Wind energy simulation framework

**Fig. 4:**
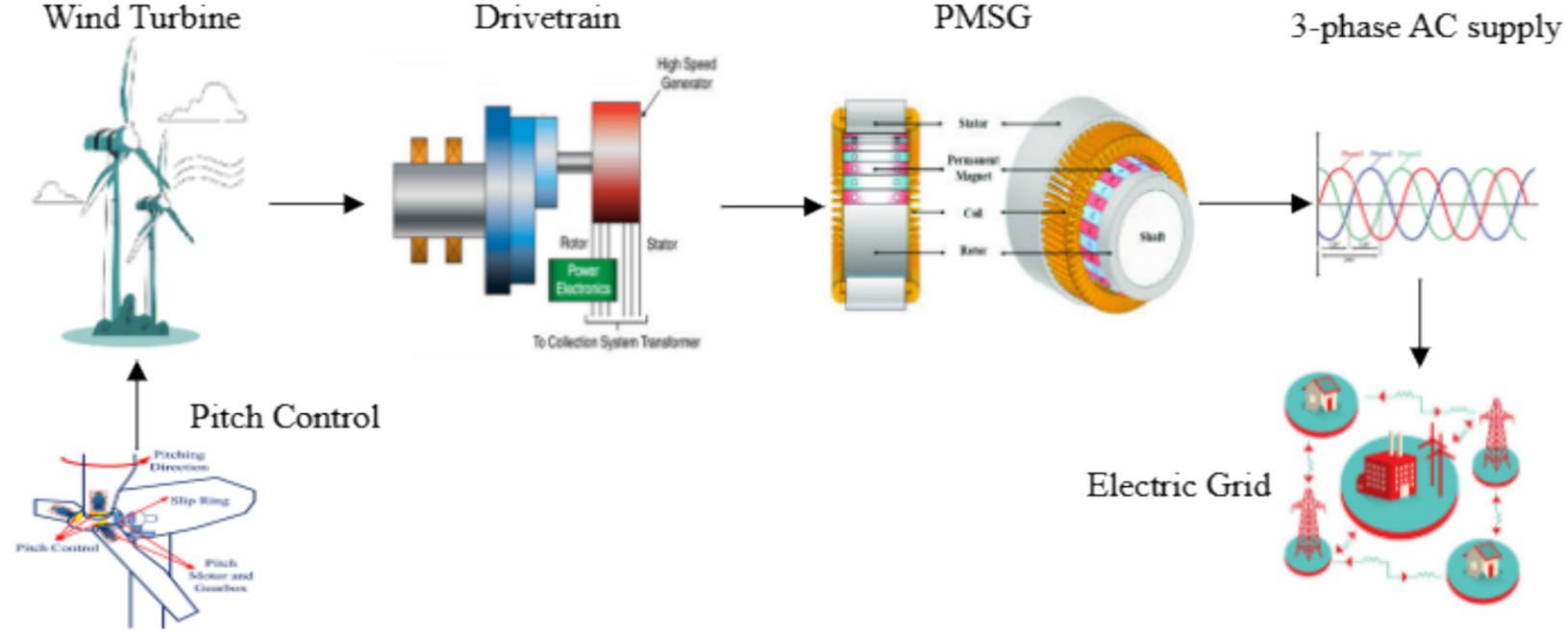
Wind Power Generation System: From Turbine to Grid—A Complete Energy Conversion and Transmission Flow Diagram.

The wind energy simulation framework, illustrated in Fig. 4, represents a comprehensive computational model that integrates all critical components of a wind power generation system to analyze and optimize energy conversion from wind resources to the electrical grid. The framework begins with aerodynamic modeling of wind turbine interactions, incorporating dynamic pitch control algorithms that continuously adjust blade angles to maximize energy capture while maintaining safety constraints across varying wind conditions. The simulation encompasses drivetrain mechanics, including gearbox efficiency, rotational dynamics, and mechanical losses during the speed conversion process from slow-rotating turbine shafts to high-speed generator inputs. The electrical conversion modeling focuses on the Permanent Magnet Synchronous Generator (PMSG) and power electronics, simulating electromagnetic interactions, voltage regulation, frequency control, and power conditioning circuits necessary for grid-compatible output. Finally, the framework incorporates grid connection modeling, which evaluates system interactions with the broader electrical network, including load balancing, power flow analysis, and considerations for grid stability. This holistic simulation approach enables engineers to predict system performance under diverse operating conditions, optimize component design and control strategies, and ensure reliable integration of renewable wind energy into existing electrical infrastructure while maintaining power quality standards and grid stability requirements.

#### Aerodynamic wind turbine model

The power equation to calculate the mechanical power output from wind turbine

9 $${\mathbf{P}}_{\mathbf{m}}=0.5\times {\varvec{\uprho}}\times \mathbf{A}\times {\mathbf{C}}_{\mathbf{p}}({\varvec{\uplambda}},{\varvec{\upbeta}})\times {\mathbf{V}}^{3}$$

where,

*V-* Wind speed in m/s,

*C*_*p*_*-* Power coefficient as a function of tip-speed ratio (λ) and pitch angle (β),

*ρ*- Air density in kg/m^3^,

A-The swept area of the wind turbine blades,

*Pm*- Output mechanical power^28^.

**Fig. 5:**
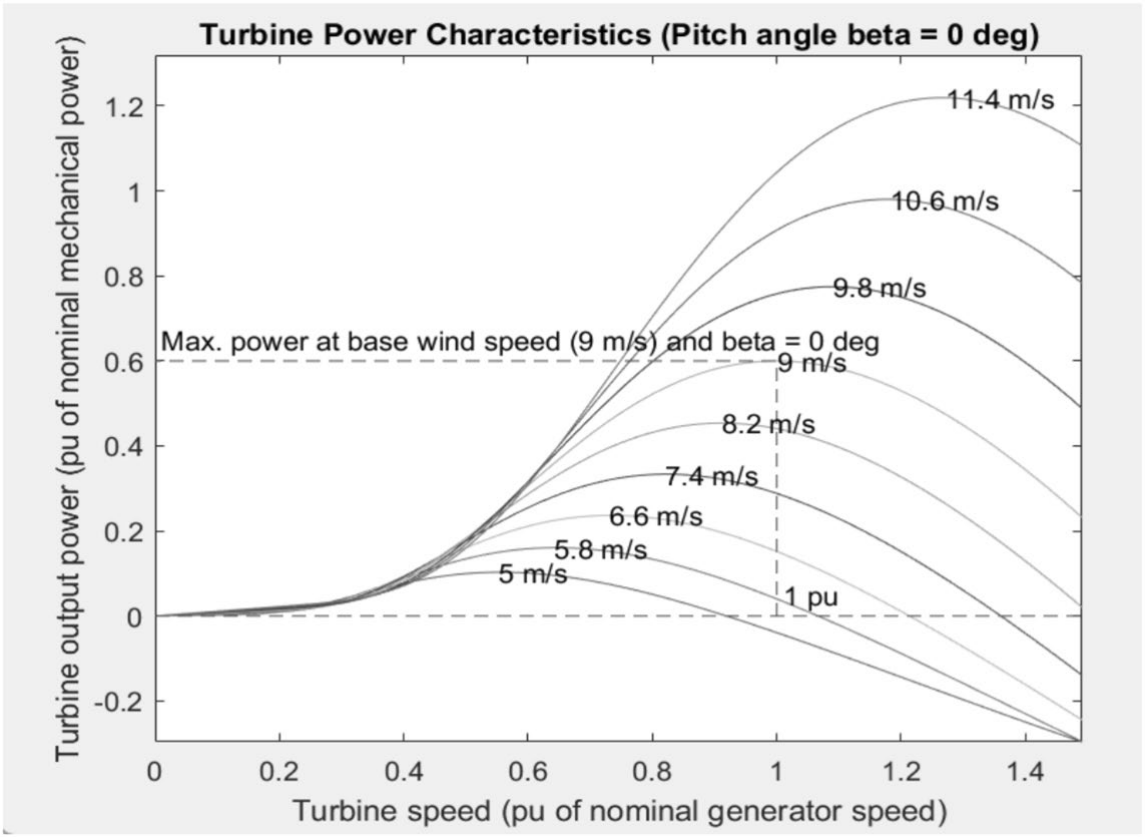
Wind Turbine Power Characteristics at Various Wind Speeds with Fixed Pitch Angle (β = 0°).

Figure 5 illustrates the relationship between turbine output power and turbine speed across a range of wind velocities, from 5 m/s to 11.4 m/s. It shows that power generation peaks at various turbine speeds, with the base wind speed of 9 m/s achieving the maximum power. Higher wind speeds result in greater power output, but they also exhibit a more pronounced decline in efficiency as turbine speeds increase.

#### Pitch control system

Pitch control in wind turbines adjusts the angle of the blades (pitch angle) to regulate power output by minimizing mechanical stress or stopping the turbine at high wind speeds to prevent damage, while also increasing aerodynamic efficiency at low to moderate wind speeds.

o At rated wind speed or higher, increase the blade pitch angle (β) to reduce Cp and limit power output.
o Control law (PID control recommended)
  10 $${\varvec{\upbeta}}={\mathbf{K}}_{\mathbf{p}}\times \left({\mathbf{P}}_{\mathbf{e}\mathbf{r}\mathbf{r}\mathbf{o}\mathbf{r}}\right)+{\mathbf{K}}_{\mathbf{i}}\times \int {\mathbf{P}}_{\mathbf{e}\mathbf{r}\mathbf{r}\mathbf{o}\mathbf{r}}\mathbf{d}\mathbf{t}+{\mathbf{K}}_{\mathbf{d}}\times \frac{{\mathbf{d}\mathbf{P}}_{\mathbf{e}\mathbf{r}\mathbf{r}\mathbf{o}\mathbf{r}}}{\mathbf{d}\mathbf{t}}$$

Where, $${{\varvec{P}}}_{{\varvec{e}}{\varvec{r}}{\varvec{r}}{\varvec{o}}{\varvec{r}}}=\boldsymbol{ }{{\varvec{P}}}_{{\varvec{r}}{\varvec{a}}{\varvec{t}}{\varvec{e}}{\varvec{d}}}-\boldsymbol{ }{{\varvec{P}}}_{{\varvec{m}}}$$^45^

#### Drivetrain model

The rotor, shaft, gearbox (if applicable), generator, and power electronics are all represented by the drivetrain model of a wind energy conversion system (WECS). Multi-mass dynamic equations are utilized to analyze torque transmission, rotational dynamics, and energy conversion efficiency, thereby optimizing performance, reliability, and fault detection in various drivetrain configurations, including geared, direct-drive, and hybrid systems.

Model the rotor, shaft, and generator dynamics. A two-mass model is commonly used:

11 $${\mathbf{J}}_{\mathbf{r}}=\frac{{\mathbf{d}\mathbf{w}}_{\mathbf{r}}}{\mathbf{d}\mathbf{t}}={\mathbf{T}}_{\mathbf{m}}-{\mathbf{T}}_{\mathbf{s}}$$

12 $${\mathbf{J}}_{\mathbf{g}}=\frac{{\mathbf{d}\mathbf{w}}_{\mathbf{g}}}{\mathbf{d}\mathbf{t}}={\mathbf{T}}_{\mathbf{s}}-{\mathbf{T}}_{\mathbf{e}}$$

where, J_r,_ J_g_- Moment of inertia for Rotor and generator, ω_r_, ω_g_- Angular velocities for Rotor and generator, T_m_- Mechanical torque from the turbine, T_s_- Shaft torque, T_e_- Electrical torque generated from the generator^46^.

#### Generator model (PMSG)

The electrical and mechanical dynamics of a wind energy conversion system (WECS) are represented by the Permanent Magnet Synchronous Generator (PMSG) model.

**Fig. 6:**
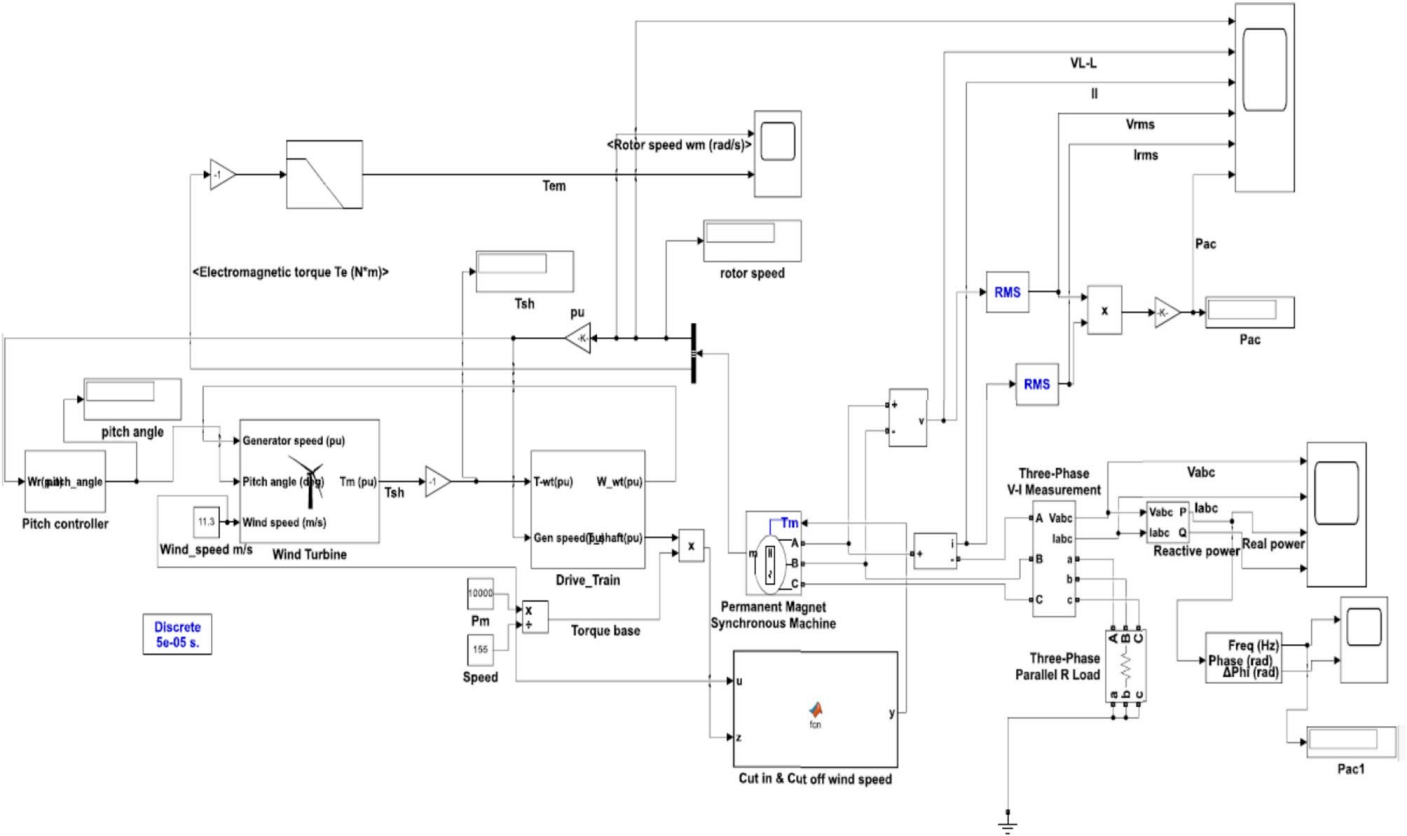
PMSG-Based Wind Turbine Control System with Drive Train and Pitch Control using predicted wind speed from the ML models.

Permanent magnets on the rotor improve efficiency and reliability by removing the need for an external excitation system, and the system is controlled by electromagnetic equations in the dq-reference frame, which are expressed as follows:

13 $${\mathbf{V}}_{\mathbf{d}}={\mathbf{R}}_{\mathbf{s}}{\mathbf{I}}_{\mathbf{d}}+{\mathbf{L}}_{\mathbf{d}}\frac{\mathbf{d}{\mathbf{I}}_{\mathbf{d}}}{\mathbf{d}\mathbf{t}}-{\varvec{\upomega}}{\mathbf{L}}_{\mathbf{q}}{\mathbf{I}}_{\mathbf{q}}$$

14 $${\mathbf{V}}_{\mathbf{q}}={\mathbf{R}}_{\mathbf{s}}{\mathbf{I}}_{\mathbf{q}}+{\mathbf{L}}_{\mathbf{q}}\frac{\mathbf{d}{\mathbf{I}}_{\mathbf{q}}}{\mathbf{d}\mathbf{t}}+{\varvec{\upomega}}{\mathbf{L}}_{\mathbf{d}}{\mathbf{I}}_{\mathbf{d}}+{\varvec{\upomega}}{{\varvec{\uplambda}}}_{\mathbf{m}}$$

15 $${\mathbf{T}}_{\mathbf{e}}=\frac{3}{2}\mathbf{P}({{\varvec{\uplambda}}}_{\mathbf{m}}{\mathbf{I}}_{\mathbf{q}}+({\mathbf{L}}_{\mathbf{d}}-{\mathbf{L}}_{\mathbf{q}}){\mathbf{I}}_{\mathbf{d}}{\mathbf{I}}_{\mathbf{q}})$$

where, V_d_, V_q_- stator voltages, I_d_, I_q_- stator currents, R_s_- stator resistance, L_d_, L_q_- stator inductances, λ_m_- flux linkage from permanent magnets, ω- electrical angular velocity, T_e_- electromagnetic torque, P- number of pole pairs. This model supports control algorithms that maximize wind energy conversion while preserving grid stability and dynamic performance, such as Field-Oriented Control (FOC) and Maximum Power Point Tracking (MPPT)^47^. Table 2 shows the values of the blocks used in building the wind energy conversion system model in MATLAB Simulink. Figure 6 illustrates a comprehensive wind turbine control system modeled in Simulink, incorporating a Permanent Magnet Synchronous Generator (PMSG) with electromagnetic torque feedback loops and wind speed inputs to optimize power generation performance. The system integrates key components, including pitch angle control, drive train dynamics, generator speed regulation, and the conversion of mechanical energy into a three-phase AC electrical output.

**Table 2:** Block parameters of the wind energy conversion system.

Block	Parameter	Value
**Turbine**	Power (W)	10,000
	Base power of Electrical generator (VA)	10,000/0.9 = 11,111
	Base wind speed (m/s)	9
	Max. power at base speed (p.u)	0.6
	Base rotational speed (p.u)	1
**Pitch Controller**	Gain	500
	Saturation limit	Upper limit = 45, Lower limit = 0
	Rate limiter	Rising slew rate = 2, Falling slew rate = −2
	Cut in & Cut off Speed (m/s)	3 & 25
**Drive train**	Wind inertia constant	0.125
	Stiffness constant	0.3
	Base wind speed (m/s)	152.8
	Damping constant	1
**PMSG**	Number of Phases	3
	Stator phase resistance Rs (Ohm)	0.425
	Armature inductance (H)	0.000835
	Flux linkage	0.53333
	Inertia (J(kg.m^2))	0.01197
	pole pairs	5
	viscous damping (F(N.m.s))	0.001189
**Load**	Resistive load (ohm)	25
**Solver**	Discrete (Fixed-step)	Ode3(Bogacki-Shampine)
**Sampling time**		50e-6

### Physical informed neural network (PINN)

**Fig. 7:**
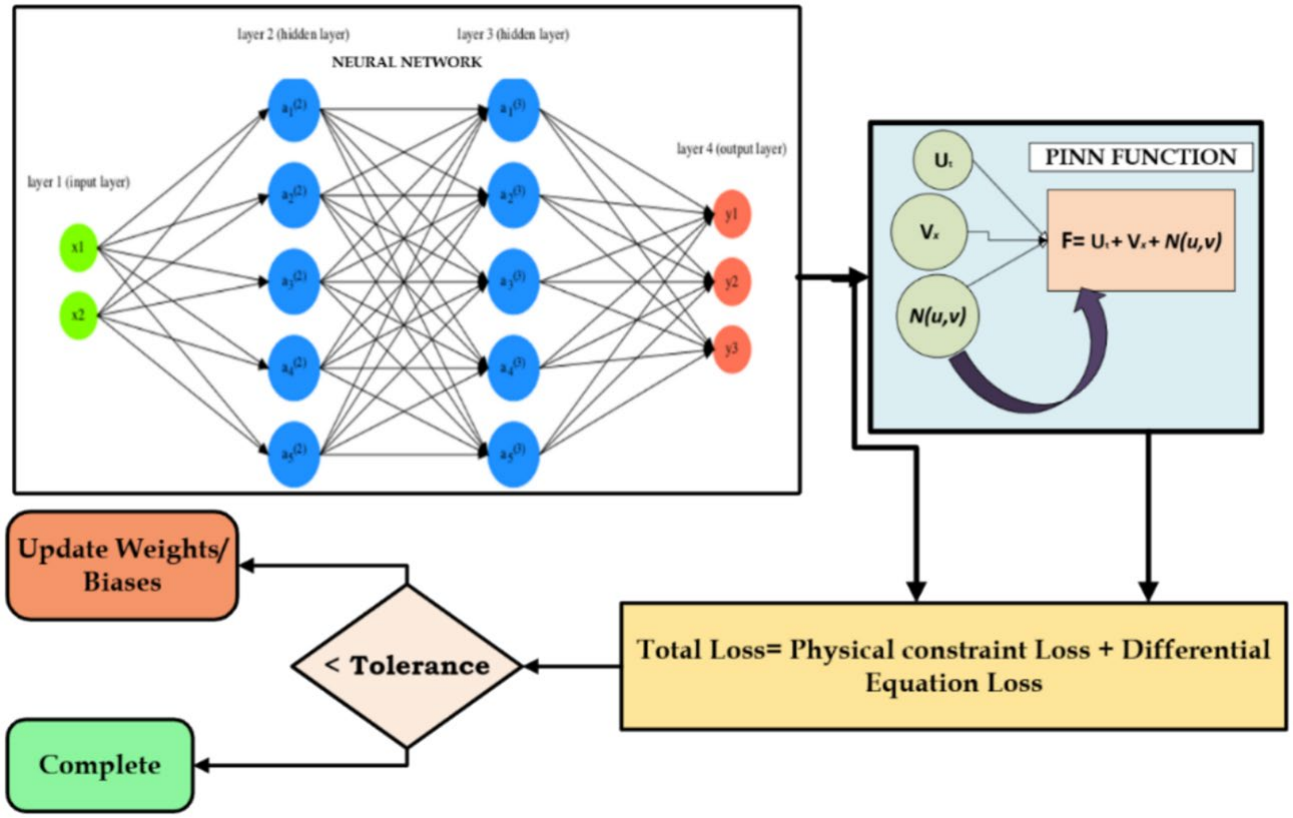
Workflow of a Physics-Informed Neural Network (PINN) for Solving Physical System Constraints.

A Physics-Informed Neural Network’s (PINN) core architecture and operational process are depicted in Fig. 7. Physical inputs are received by the neural network, which translates them into output variables. It is composed of an input layer, several hidden layers, and an output layer. In contrast to traditional neural networks, PINNs use a loss function that blends differential equation loss with physical constraint loss to incorporate physical information. This ensures that the anticipated results comply with the governing physical laws, in addition to aligning with the training data. The weights and biases are finalized when the overall loss drops below a predetermined tolerance, which is achieved by continuing the training cycle. By bridging the gap between physics-based systems and data-driven models, this hybrid learning technique makes PINNs ideal for intricate scientific and engineering applications.

#### Mathematical modelling of PINN

To model electricity generation $\widehat{P}\left(t\right)$ From wind energy using a neural network that:

- Learns from real-time measurements
- Obeys physical constraints (from simulation)
- Enforces power-wind relationships and turbine characteristics
- **Inputs and features**

Let,

v(t)= Wind speed at time t (from real data)

θ(t)= Pitch angle (from simulation)

*w*_*r*_(t)= Rotor speed (from simulation)

T_m_(t)= Mechanical torque (from simulation)

T_e_(t)= Electrical torque (from simulation)

X(t)∈ℝ^d^= Complete feature vector including time-based cyclic features

16 $$X\left( t \right)\, = \,[v\left( t \right),\theta \left( t \right),w_{r} \left( t \right), \, T_{m} \left( t \right), \, T_{e} \left( t \right), \, \sin (\frac{2\Pi Month}{{12}}), \, \cos (\frac{2\Pi Month}{{12}}), \ldots ..]$$

- Neural network approximation

f_θ_= Neural network with parameters θ

$\widehat{P}\left(t\right)$= Predicted electrical power output at time t.

- Data loss (supervised learning term)
  17 $$L_{data} = \frac{1}{N}\sum\nolimits_{(i = 1)}^{N} {(\hat{P}(t_{i} ) - Pmeasured(t_{i} ))^{2} }   $$
- **Physics-based loss (from MATLAB simulink model)**
- **Wind power equation**
  18 $${\mathbf{P}}_{\mathbf{m}}(\mathbf{t})=0.5\times {\varvec{\uprho}}\times \mathbf{A}\times {\mathbf{C}}_{\mathbf{p}}({\varvec{\uplambda}},{\varvec{\upbeta}})\times {\mathbf{V}}^{3}$$

Where,

*V-* Wind speed in m/s,

*C*_*p*_*-* Power coefficient as a function of tip-speed ratio (λ) and pitch angle (β),

*ρ*- Air density in kg/m^3^,

A-The swept area of the wind turbine blades,

*P*_*m*_- Output mechanical power. Using Betz’s limit,

19 $$P_{expected} \left( t \right)\, = \,\eta \times P_{m} (t)$$

Typical efficiency= 0.6

- Constraint: Predicted power must not exceed expected power
  20 $$Lefficiency = \frac{1}{N}\sum\nolimits_{(i = 1)}^{N} {[\max (0,\hat{P}(t_{i} ) - \eta \times P_{m} (t_{i} ))]^{2} }$$
- Cut-in, rated, and cut-out constraint

Cut- in speed *V*_in_= 3 m/s

Rated speed *V*_rated_= 9 m/s

Cut- out speed *V*_out_= 25 m/s

η(*V*) = $$\left\{\begin{array}{c}0 V< {V}_{in} or V> {V}_{out}\\ 1 otherwise\end{array}\right.$$

**Constraints:**

21 $$Lcutoff = \frac{1}{N}\sum\nolimits_{(i = 1)}^{N} {[\hat{P}(t_{i} ) \times } (1 - \eta (V_{i} )]^{2}$$

- Rotor- Machine relationship Constraint

From physics and simulation:

22 $$P_{sim} (t) = w_{r} (t) \times T_{e} (t)$$

So enforce:

23 $$L_{s} im = \frac{1}{N}\sum\nolimits_{(i = 1)}^{N} {(\hat{p}(t_{i} )} - P_{s} im(t_{i} ))^{2}$$

- Total Loss Function (Physics-Informed)
  24 $$L_{total} = L_{data} + \alpha_{1} L_{efficiency} + \alpha_{2} L_{cutoff} + \alpha_{3} L_{sim}$$

α_i-_ *Tunable weights for each physical constraint.*

#### Dataset configuration for PINN model evaluation

This section outlines the comprehensive dataset strategy employed for PINN model evaluation, which leverages a dual-source data approach to maximize both empirical accuracy and physical consistency. The evaluation framework employs a hybrid data integration strategy to train and validate the PINN model by combining real-world and simulation-based datasets. The real-time MERRA-2 dataset (Case 1) provides globally consistent meteorological inputs, including date, time, wind speed, and electricity generation (in kW). To complement this, a synthetic dataset is generated through MATLAB-based physical simulations (Case 2), where partial differential equations governing wind turbine aerodynamics and electromechanical behavior are used to compute critical operational parameters such as pitch angle, rotor speed, mechanical torque, electrical torque, and generated power. By integrating these two data sources, the PINN model can simultaneously learn from empirical observations and the governing physical laws of wind energy conversion. This approach ensures that predictions are not only data-driven but also physically consistent, effectively capturing the complex, real-world dynamics essential for accurate and reliable wind power forecasting.

Table 3 presents the performance analysis across varying wind speeds, illustrating the characteristic operational behaviour of wind turbines under different atmospheric conditions and revealing distinct performance zones based on wind velocity. At the above-rated wind speed of 14 m/s, the turbine operates with active pitch control (1.072 p.u) to regulate power output, achieving high rotor speed (153.1 rad/s), near-optimal mechanical torque (0.9979 Nm), substantial electrical torque (64.2 Nm), and maximum electricity generation (9788.98 W). The rated wind speed condition of 9 m/s represents optimal operation, where the turbine maintains efficient performance with zero pitch angle adjustment, a good rotor speed (133.3 rad/s), moderate mechanical torque (0.7216 Nm), and a substantial power output (7386.1 W). In contrast, the below-rated wind speed scenario of 6 m/s illustrates sub-optimal operation where the turbine experiences reduced efficiency with minimal mechanical torque (0.2531 Nm), lower rotor speed (95.48 rad/s), and significantly decreased electricity generation (3806.93 W), highlighting the direct correlation between wind speed availability and turbine performance parameters across the operational envelope. The analysis reveals that electricity generation varies dramatically with wind speed, following the cubic relationship P ∝ V^3^, where power output increases from 3806.93 W at 6 m/s to 9788.98 W at 14 m/s. This demonstrates how wind velocity directly governs energy conversion efficiency, making accurate wind resource assessment critical for reliable renewable energy planning and grid integration.

**Table 3:** Wind Turbine Performance Parameters at Different Wind Speed Operating Conditions.

Wind speed (m/s)	Pitch angle (p.u)	Rotor speed (rad/s)	Mechanical Torque (Nm)	Electrical Torque (Nm)	Electricity generated(W)
14	1.072	153.1	0.9979	64.2	9788.98
9	0	133.3	0.7216	55.91	7386.1
6	0	95.48	0.2531	40.05	3806.93

## Results and discussion

The results and discussion section presents a comprehensive analysis of three distinct cases designed to evaluate and compare the effectiveness of machine learning (ML), physics-based modeling, and Physics-Informed Neural Networks (PINNs) in forecasting wind power generation.

### Case 1: Machine learning models for wind energy prediction

Table 4 presents the performance evaluation of various machine learning models applied to a regression task, assessed using MAE, MSE, RMSE, and the R^2^ on both training and testing datasets. Ensemble methods, including XGBoost, Gradient Boosting, Random Forest, Extra Trees, Decision Trees, and Stacking models, showed outstanding performance, achieving low error metrics and high R^2^ values (above 0.99), indicating excellent predictive accuracy. The Stacking ensemble model emerged as the best performer, with the lowest testing RMSE (0.11) and near-perfect R^2^ (0.998). AdaBoost performed well but was outperformed by other boosting methods. KNN, LR, and NN showed higher error rates and lower R^2^ scores (0.931 to 0.967), suggesting weaker data fit. While Random Forest and XGBoost maintained a good balance between training and testing performance, indicating minimal overfitting, Decision Tree and Extra Tree showed near-perfect training scores with lower testing performance, suggesting minor overfitting. Ensemble-based approaches, particularly boosting and bagging methods, proved to be the most effective for the regression problem.

**Table 4:** Regression Model Performance Metrics on Training and Testing Data.

MODEL	MAE		MSE		RMSE		R^2^	
	**Testing**	**Training**	**Testing**	**Training**	**Testing**	**Training**	**Testing**	**Training**
**AdaBoost**	0.194	0.19	0.073	0.06	0.271	0.246	0.986	0.989
**Decision Tree**	0.03	0.01	0.038	0.02	0.194	0.01	0.993	0.998
**Extra Tree**	0.03	0.01	0.027	0.02	0.163	0.049	0.995	0.997
**Gradient Boosting**	0.035	0.032	0.017	0.01	0.131	0.097	0.997	0.998
**K-Nearest Neighbours**	0.295	0.225	0.176	0.107	0.42	0.327	0.967	0.98
**Linear Regression**	0.481	0.473	0.349	0.34	0.591	0.583	0.935	0.937
**Neural Network**	0.463	0.457	0.372	0.364	0.61	0.603	0.931	0.933
**Random Forest**	**0.027**	**0.009**	**0.026**	**0.002**	**0.16**	**0.047**	**0.995**	**0.999**
**XGBoost**	**0.035**	**0.014**	**0.014**	**0.001**	**0.119**	**0.026**	**0.997**	**0.999**
**Stacking Ensemble (RF + XGB)**	**0.026**	**0.012**	**0.013**	**0.001**	**0.11**	**0.024**	**0.998**	**0.999**

**Fig. 8:**
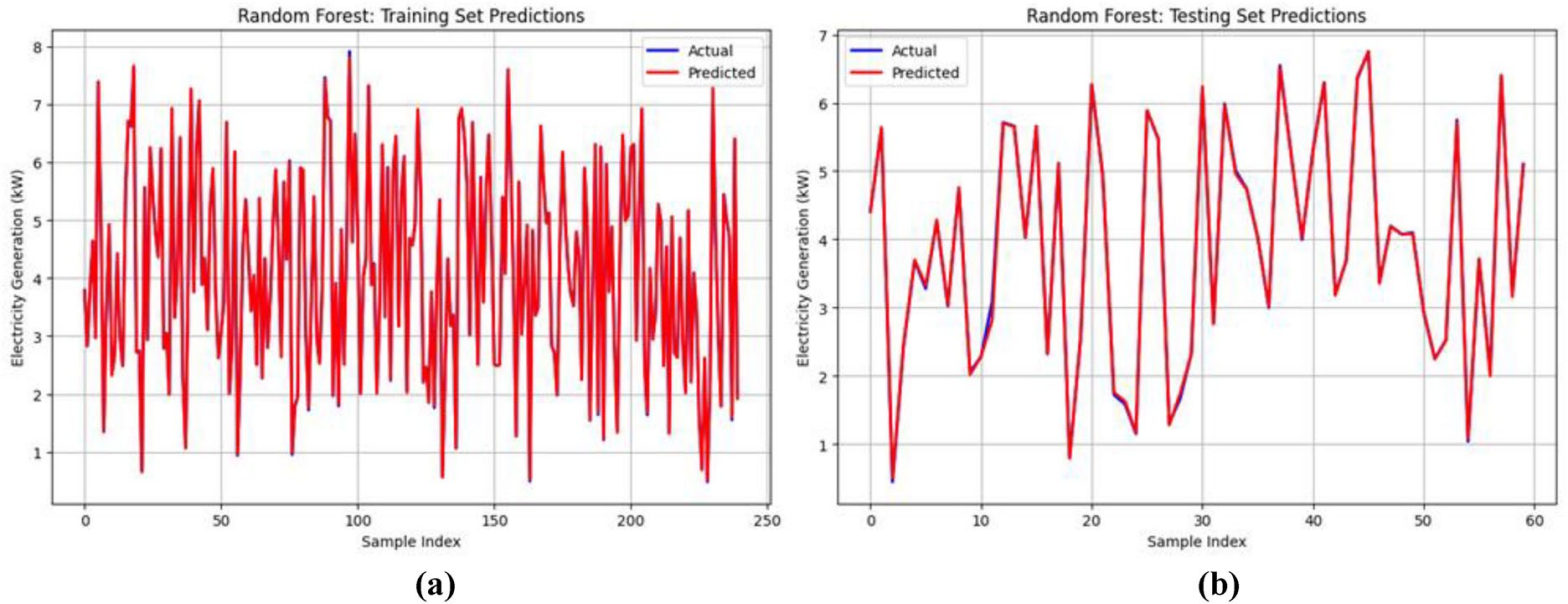
Prediction of the Random Forest model on the training and testing datasets.

**Fig. 9:**
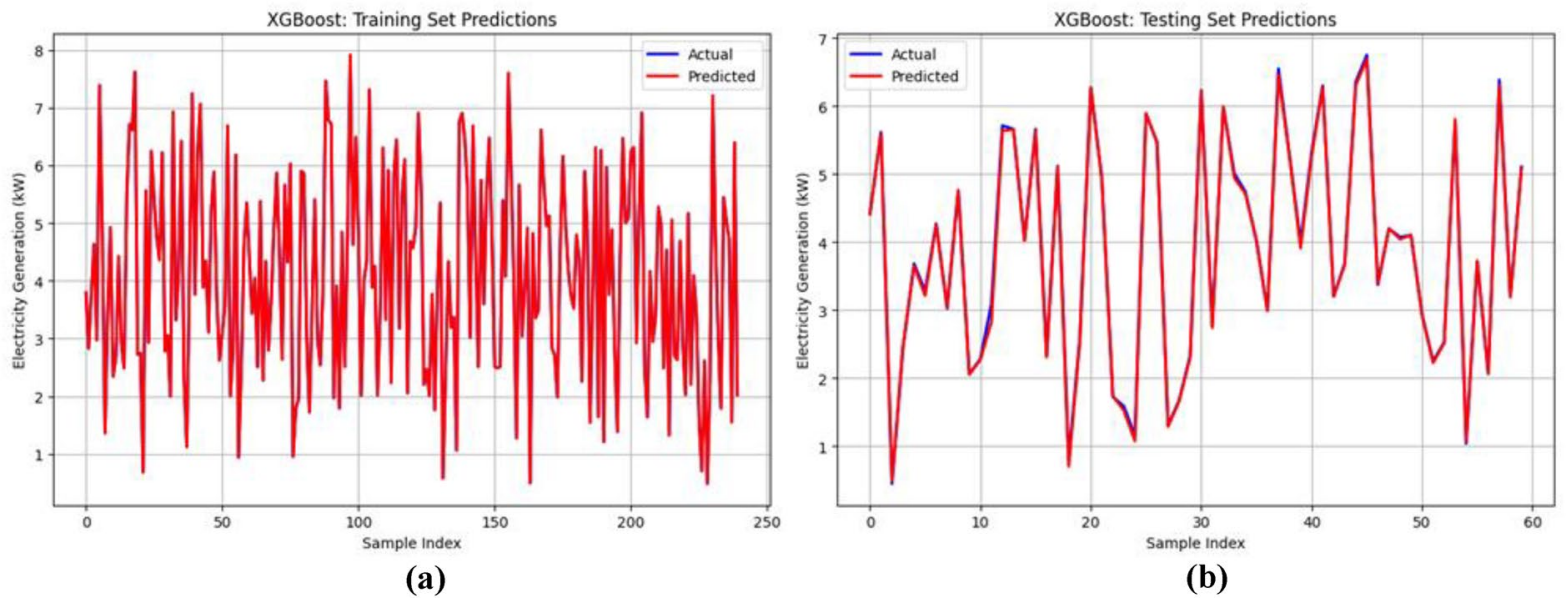
Prediction of the XGboost model on the training and testing datasets.

**Fig. 10:**
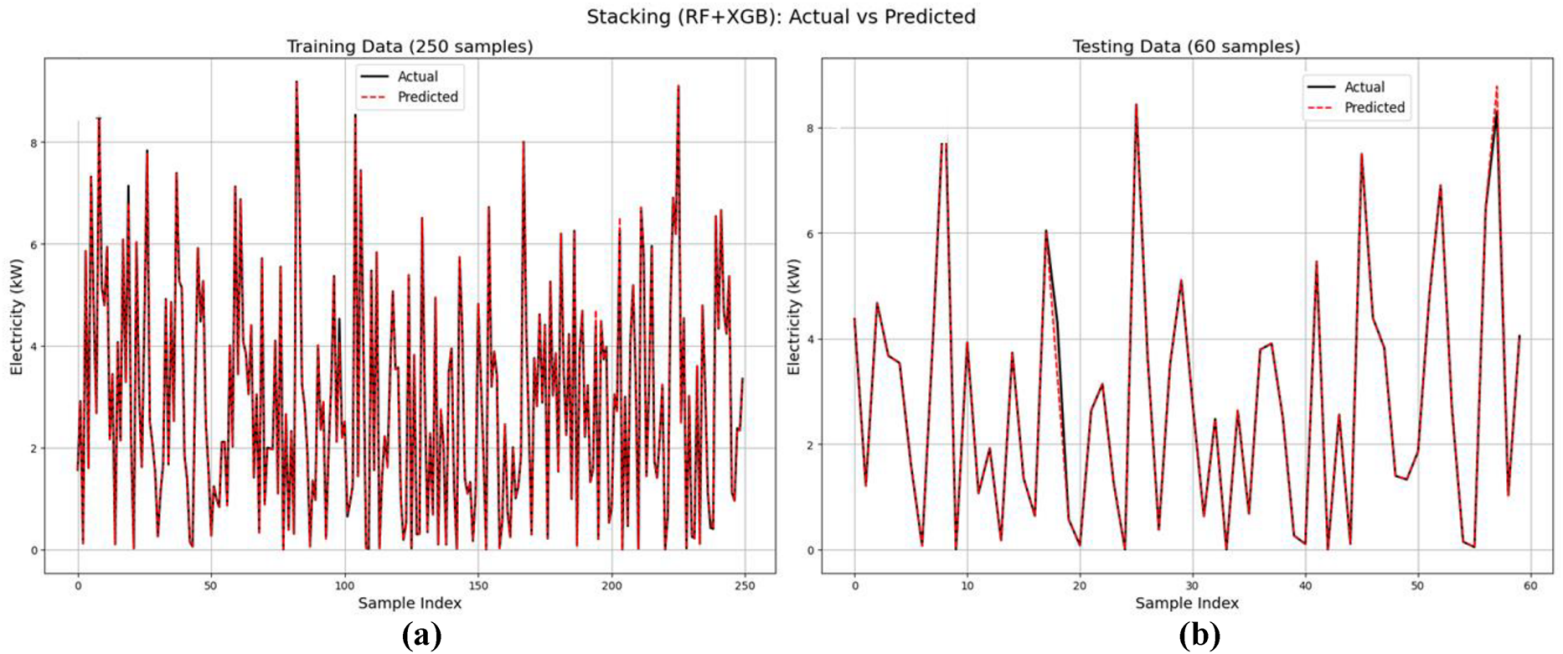
Prediction of the Stacking ensemble model on the training and testing datasets.

From Table 4, it is observed that the Random Forest, XGBoost, and Stacking ensemble models perform well in both the training and testing datasets. Therefore, for further analysis, these three models were taken for the evaluation. From Fig. 8, the training set (Fig. 8(a)) shows high prediction accuracy, with aligned actual and predicted values across 250 samples. The testing set (Fig. 8(b)) shows prediction deviations across 60 samples, suggesting model generalization challenges in wind power forecasting. The plots compare the Random Forest model’s predictions with actual values for both the training and testing datasets. Figure 9 illustrates the predictive performance of the XGBoost model. The training set (Fig. 9(a)) displays dense predictions across 250 samples, covering electricity generation ranges from 1 to 8 MW. The testing set (Fig. 9(b)) demonstrates model generalization across 60 samples with values from 1 to 7 MW. Although prediction patterns differ, a close alignment exists between the actual (blue) and predicted (red) values. Figure 10 shows actual versus predicted electricity generation (in kW) using the Stacking ensemble model (combining Random Forest and XGBoost) for training and testing datasets. Figure 10(a) shows training data (250 samples) exhibits a close match between actual and predicted values, indicating an excellent model fit. Figure 10(b) shows testing data (60 samples) demonstrating excellent agreement between actual and predicted outputs, with minimal deviation. This suggests that the Stacking model generalizes well and is highly effective in predicting electricity generation from wind speed data.

### Case 2: Physical model for 10 kW Wind Turbine in MATLAB simulink

**Fig. 11:**
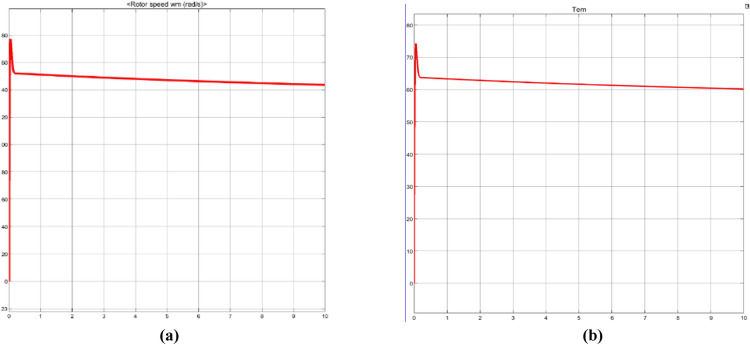
Rotor Speed and Electromechanical Torque Transient Response.

In Fig. 11, the two plots (Fig. 11(a) and (b)) show the dynamic properties of a wind turbine system over a 10-s simulation. The rotor speed (wm rad/s) shows a sharp initial decline from 80 rad/s, followed by a gradual stabilization at 40 rad/s. In contrast, the electromagnetic torque (Tem) shows a similarly rapid drop from its peak of 75 Nm to a steady-state value of 60 Nm. This shows the damped response of the system and the inherent coupling between rotational dynamics and electromagnetic forces during the transient phase.

**Fig. 12:**
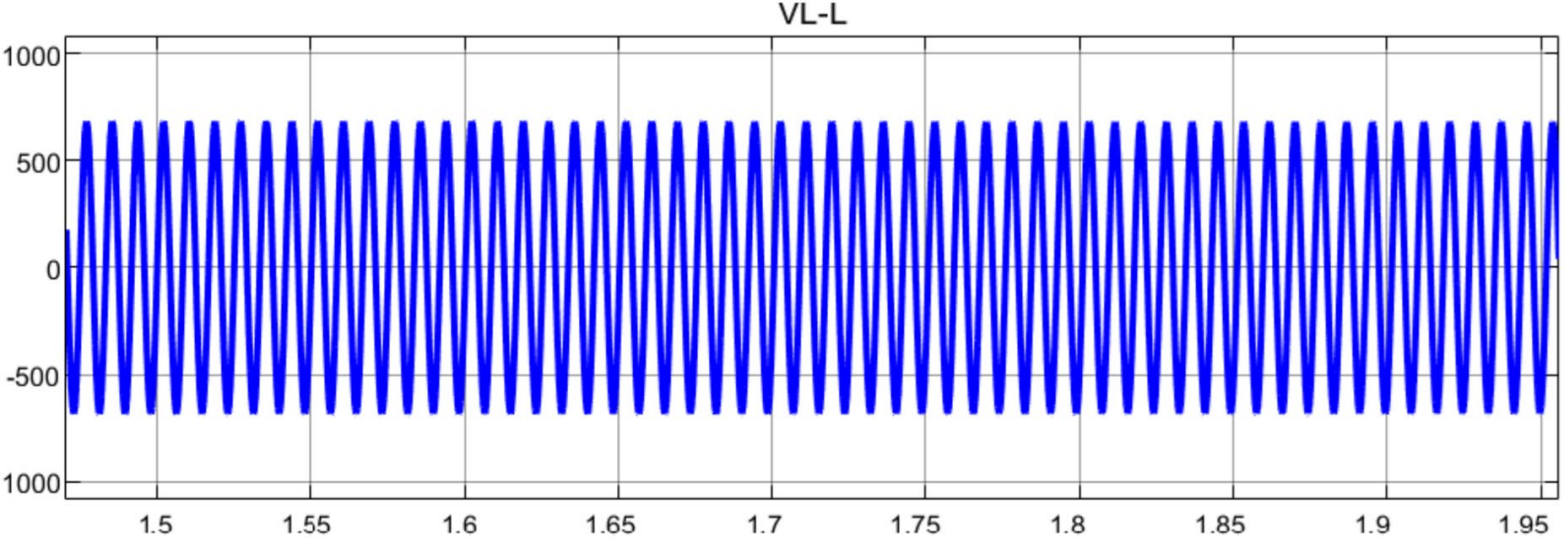
Line-to-Line Voltage Waveform Analysis.

Figure 12 shows the wind turbine’s line-to-line voltage (VL-L) output waveform with a peak-to-peak value of around ± 700 V, and the waveform displays a steady sinusoidal pattern, suggesting steady voltage production. Throughout the 1.5 to 1.95 s measuring period, the signal’s amplitude remains constant, indicating consistent power production. Within the 0.45-s interval, the waveform displays almost 45 full cycles, which corresponds to a frequency of roughly 100 Hz. This indicates that the system is operating steadily and at the regular grid frequency parameters. The signal displays:

- A clear sinusoidal form devoid of noticeable distortion
- A steady amplitude free of voltage surges or sags
- Consistent frequency with negligible variations
- Equilibrium voltage levels in the half-cycles of positive and negative

These traits indicate good grid synchronization and an effective power conversion system, both of which are essential for integrating wind power into the electrical grid.

**Fig. 13:**
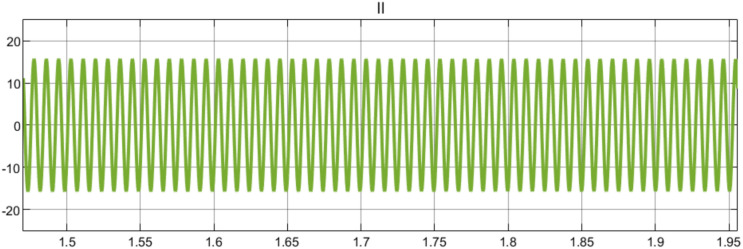
Phase Current Waveform Analysis.

Under normal operating conditions, Fig. 13 shows a steady-state phase current waveform oscillating between + 15 A and −15A for 1.5 to 1.95 s. It has a uniform sinusoidal pattern with consistent amplitude and frequency, indicating balanced operation of the wind turbine’s electrical system and stable current flow. The stability and homogeneity of the current waveform indicate appropriate operation of the power conversion system and efficient current regulation, both of which are necessary for reliable wind power generation.

**Fig. 14:**
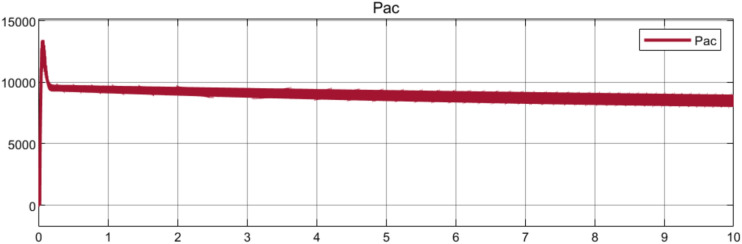
Active Power Output Response.

**Fig. 15:**
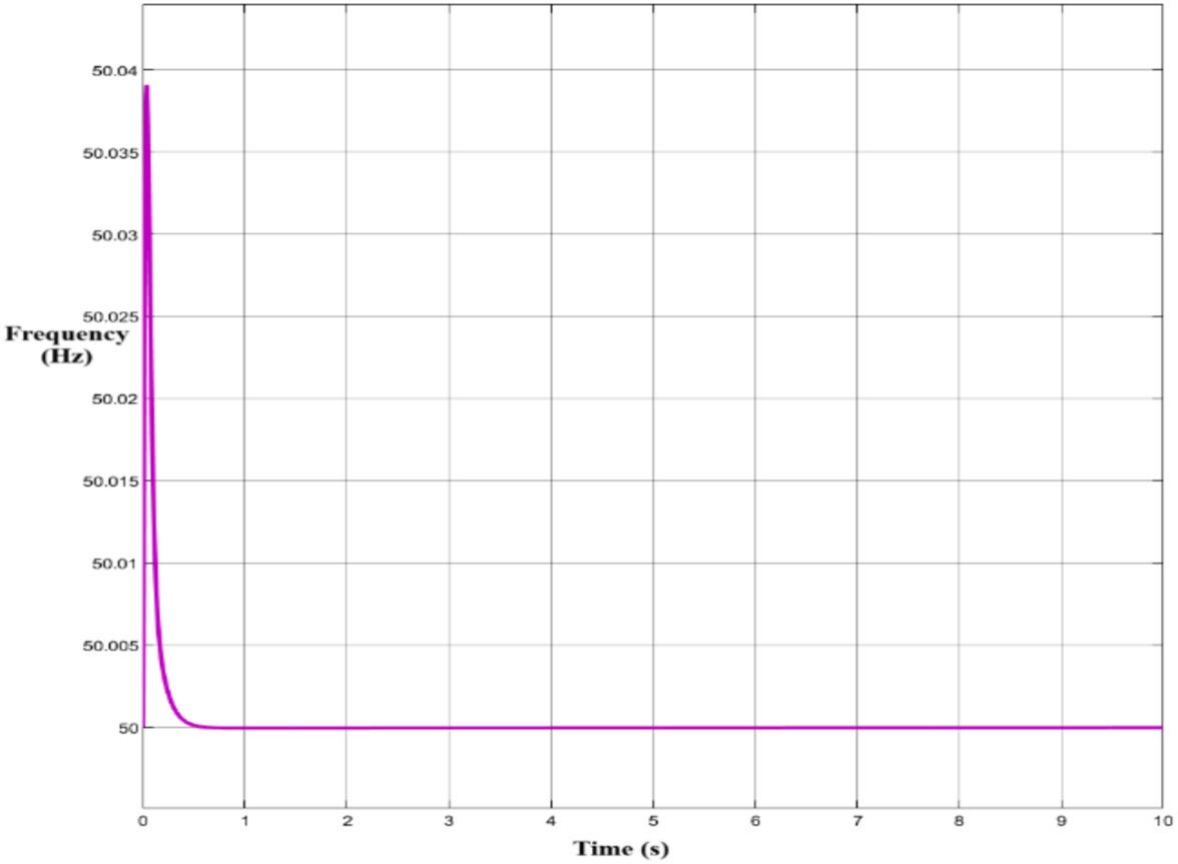
Grid Frequency Response Analysis.

Figure 14 displays the active power (Pac) output of a wind turbine system over a 10-s simulation. It shows an initial power surge of approximately 13 kW, followed by a rapid stabilization to around 9 kW. The system then maintains a steady power output with few fluctuations, indicating that the power control system is effective in achieving stable operation. To demonstrate the system’s efficient frequency regulation and return to steady-state operation following a brief disturbance, Fig. 15 shows the dynamic behaviour of grid frequency over 10 s. Initial frequency spikes to 50.03 Hz are followed by rapid decay and stabilization at the nominal frequency of 50 Hz.

#### Performance evaluation of physical model in matlab simulink: computational and dynamic insights

The physical model simulates a wind energy conversion system (WECS) that incorporates a turbine, pitch controller, drive train, permanent magnet synchronous generator (PMSG), and a resistive load. A discrete fixed-step solver (Ode3-Bogacki-Shampine) with a sampling time of 50 µs is used to implement the system in MATLAB Simulink. This assures high-resolution numerical accuracy, which is crucial for capturing the rapid dynamics of power electronic and electromechanical systems.

Key system parameters indicate a moderately realistic and detailed portrayal:

- The turbine’s reasonable efficiency assumption (0.9) is reflected in its 10-kW mechanical power and 11.1 kVA base electrical output.
- Under variable wind conditions, the pitch controller is designed to actively manage turbine output and prevent overspeed (cut-in at 3 m/s, cut-off at 25 m/s). It has a high gain (500) and strict saturation and rate limitations.
- Standard mechanical characteristics (inertia = 0.125, damping = 1, stiffness = 0.3) control the drive train dynamics, and a base wind speed mismatch (152.8 m/s) could be a typo or a symbolic representation.
- Generator behavior under dynamic wind input is accurately reflected by the PMSG’s specific electromagnetic parameters (Rs = 0.425 Ω, inductance = 0.000835 H, flux linkage = 0.53333 Wb), mechanical inertia (0.01197 kg·m^2^), and damping (0.001189 N·m·s).
- The grid interface is made simpler, and internal dynamics are highlighted by utilizing a 25-Ω resistive element as the load.

Table 5 reveals a clear positive correlation between wind speed and execution time in what appears to be a computational or processing system. At the lowest wind speed of 3 m/s, the execution time is approximately 11.05 s, representing the baseline performance. As wind speed increases to 6 m/s and 9 m/s, there is a modest but noticeable increase in execution time to 11.14 and 11.19 s, respectively, suggesting a gradual degradation in system performance. However, the most significant impact occurs at higher wind speeds, where execution time jumps dramatically to 13.07 s at 11.3 m/s and reaches 13.12 s at 14 m/s wind speed. This pattern indicates that the system maintains relatively stable performance under moderate wind conditions but experiences substantial performance degradation when wind speeds exceed approximately 11 m per second. The nearly 2-s increase in execution time between low and high wind conditions represents a roughly 18% decrease in performance, suggesting that wind speed is a critical environmental factor affecting the system’s operational efficiency. This relationship can be attributed to various factors, such as mechanical vibrations, cooling system efficiency, or environmental interference, that affect the processing equipment. For hardware-in-loop or control development applications, the fixed-step discrete solver ensures numerical stability, and high-fidelity response analysis is enabled by comprehensive component modeling. As a result, the model can be used for control validation, performance evaluation, and comparison with data-driven models such as ML-based forecasters or PINNs.

**Table 5:** Wind Speed Impact on Computational Execution Time.

Wind Speed (m/s)	Execution Time (seconds)
3	11.0459
6	11.1447
9	11.1897
11.3	13.0657
14	13.1237

Table 6 presents a comprehensive justification for the validation of each wind turbine parameter simulated in MATLAB Simulink. The cut-in speed of 3 m/s aligns with the typical operational range for small to medium turbines as outlined by^29^, providing theoretical backing from established design standards. The rated speed of 9 m/s and cut-out speed of 25 m/s both correspond with the NREL benchmark values reported by Hansen et al. (2004), confirming that the turbine operates within expected wind speed thresholds. The rated power output of 10.0 kW also directly aligns with the NREL specifications, indicating the correct sizing and scaling of the simulated system. The power coefficient (Cp) of approximately 0.41 at a tip-speed ratio of 8 is within the efficient range of 0.40–0.45 described by^29^, demonstrating aerodynamic efficiency. Lastly, the torque-speed curve generated in the simulation aligns with the theoretical behavior of the d-q axis. It matches the MATLAB-based PMSG simulation results from^46^, validating the control and electrical modeling of the generator.

**Table 6:** Quantitative Validation of Simulink-Based WECS Model Against Benchmark Wind Turbine Performance Metrics.

Metric	Simulink Output	Benchmark Range	Validation Status	References
Cut-in speed	3 m/s	3.0–4.0 m/s	Matches range	Manwell et al^29^.
Rated speed	9 m/s	9–11.0 m/s	Matches range	Hansen et al. (2004)^28^
Cut-out speed	25 m/s	25 m/s (standard)	Exact match	
Rated power output	10.0 kW	10.0 kW (benchmark)	Exact match	
Power coefficient (Cp)	~ 0.41 (at λ = 8)	0.40–0.45	Acceptable range	Manwell et al^29^.
Torque-speed relationship	Matches d-q theory curve	Matches the NREL baseline	Matches shape	Yang et al^46^.

### Case 3: PINN model for wind energy prediction

In this study, a sophisticated implementation of the Physics-Informed Neural Network (PINN) model is presented, which leverages the synergistic combination of high-quality real-time meteorological data and physics-based synthetic datasets to achieve robust wind power prediction.

The PINN architecture uses the dataset as described in Sect."Dataset Configuration for PINN Model Evaluation". The synthetic data generation process, rooted in fundamental differential equations governing wind turbine dynamics, systematically encompasses critical operational parameters including mechanical torque variations, electrical torque responses, adaptive pitch angle controls, rotor speed fluctuations, precise cut-in and cut-off speed thresholds, and angular velocity dynamics across different wind speed scenarios. This comprehensive data fusion approach enables the PINN model to learn not only from observed real-world patterns but also from the underlyseecing physical principles that govern wind energy conversion, ensuring that predictions remain consistent with established laws in aerodynamic and electrical engineering. The implementation demonstrates the model’s capability to handle diverse operational scenarios while maintaining physical consistency, making it particularly valuable for real-time wind farm optimization and predictive maintenance applications where both accuracy and physical interpretability are essential.

Table 7 presents a comprehensive performance comparison between the proposed models and benchmark results from Abdelsattar etal^48^., evaluating wind power prediction accuracy across different datasets using standard regression metrics including Mean Absolute Error (MAE), Mean Squared Error (MSE), Root Mean Squared Error (RMSE), and Coefficient of Determination (R^2^). The benchmark study by Abdelsattar etal^48^. utilized a Kaggle dataset containing 40,000 observations with comprehensive environmental variables including temperature, relative humidity, dew point temperature, wind speed at multiple heights (10 m and 100 m), wind direction, wind gusts, and power output for wind turbine forecasting. Their extensive evaluation of multiple machine learning models (Random Forest, CatBoost, XGBoost, LightGBM, Support Vector Regressor, Linear Regression, and AdaBoost) and deep learning architectures (CNN, RNN, ANN, and LSTM) identified the Extra Trees algorithm as the best-performing ML model with an R^2^ of 0.7231 and RMSE of 0.1512. At the same time, the ANN achieved the highest performance among deep learning models with an R^2^ of 0.7248 and RMSE of 0.6551. In contrast, the proposed Stacking Ensemble and PINN models demonstrate superior performance on both the same Kaggle dataset and the real-time MERRA-2 dataset, with the Stacking Ensemble achieving exceptional results on MERRA-2 data (MAE: 0.026, RMSE: 0.11, R^2^: 0.998) and both proposed models significantly outperforming the benchmark models across all evaluation metrics, highlighting the effectiveness of advanced ensemble techniques and physics-informed approaches for wind power prediction.

**Table 7:** Comparative Performance Analysis of Proposed Wind Power Prediction Models Against Benchmark Results.

Dataset	Model	MAE	MSE	RMSE	R^2^
		**Testing**	**Testing**	**Testing**	**Testing**
**Kaggle dataset**	**Extra Tree (**Abdelsattar, M., et al. (2025))^48^	-	-	0.1512	0.7231
	**ANN (**Abdelsattar, M., et al. (2025))^48^	-	-	0.6551	0.7248
	**Stacking Ensemble** (Proposed model)	0.0759	0.0112	0.1058	0.8643
	**PINN** (Proposed model)	0.0872	0.0136	0.1165	0.8356
**Real-Time MERRA-2 dataset**	**Stacking Ensemble** (Proposed model)	0.026	0.013	0.11	0.998
	**PINN** (Proposed model)	0.1629	0.0586	0.2420	0.989

**Fig. 16:**
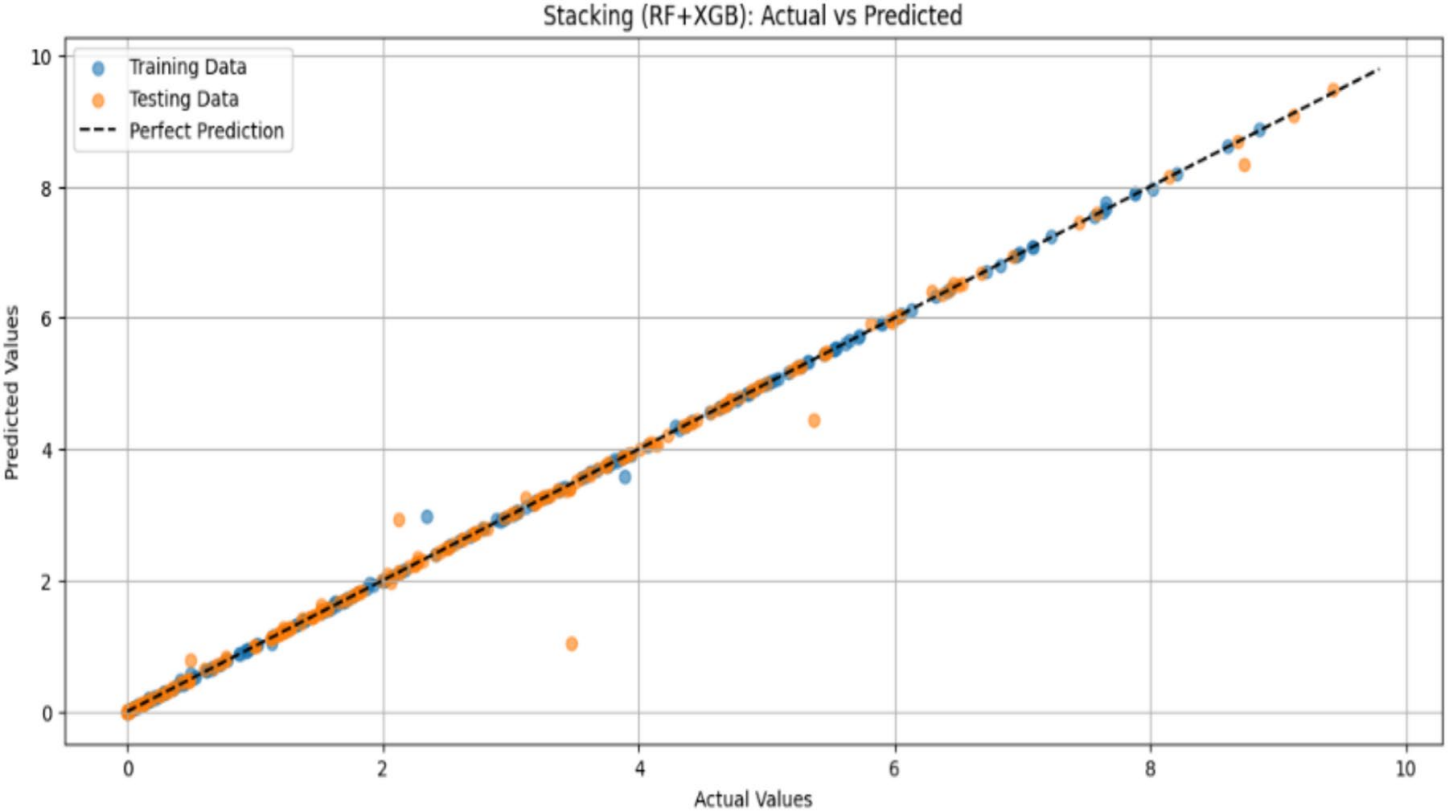
High-Fidelity Prediction Using Stacked Ensemble Model: Actual vs Predicted Values.

**Fig. 17:**
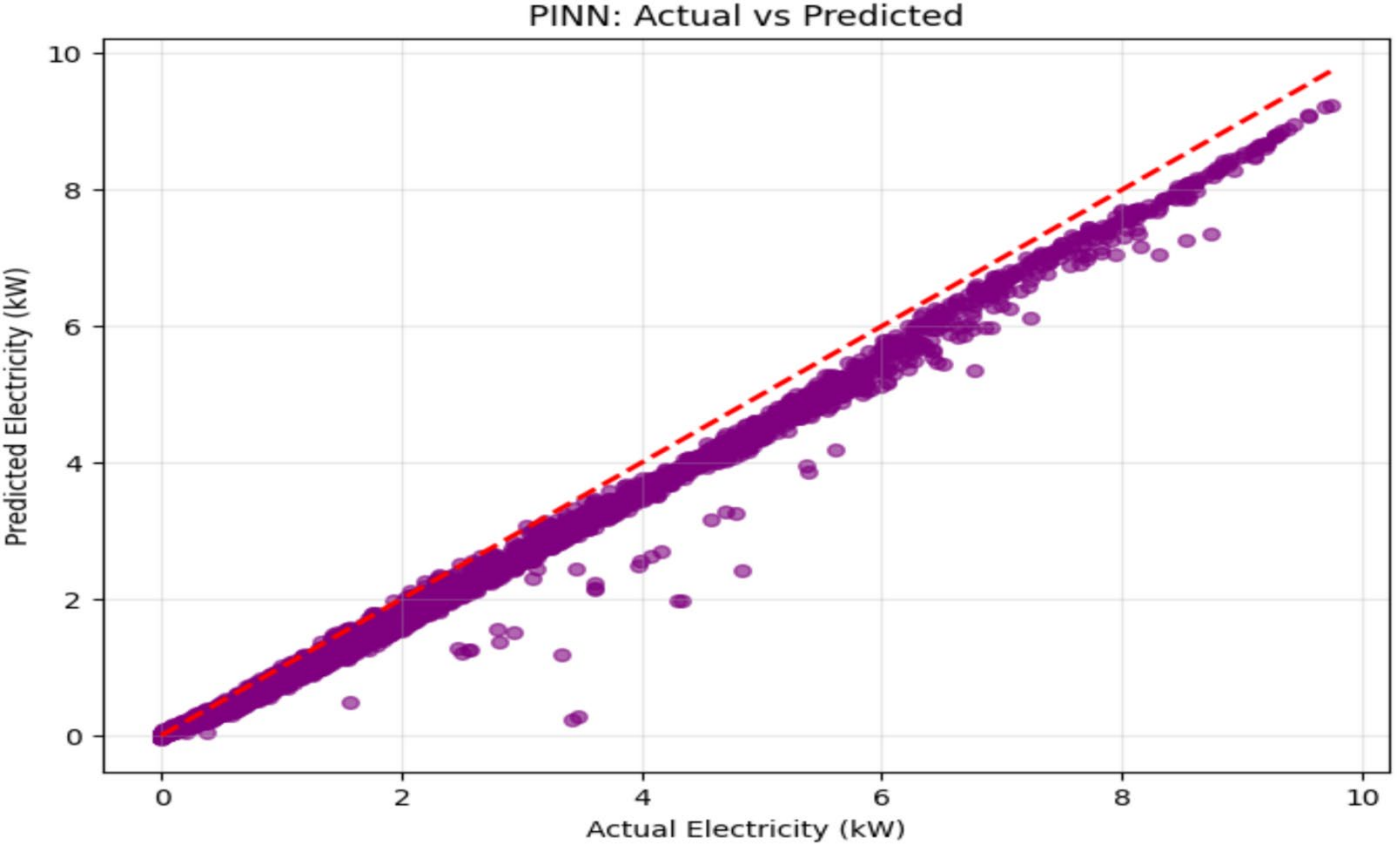
Electricity Prediction Performance of Physics-Informed Neural Network (PINN): Actual vs Predicted Values.

Figure 16 illustrates the prediction performance of the Stacking ensemble model, which combines Random Forest (RF) and XGBoost (XGB). The projected values demonstrate strong model accuracy for both the training and testing datasets, which closely align with the optimal 45-degree diagonal line. The line’s small scatter indicates a low prediction error. On the testing dataset, the model quantitatively obtained a high coefficient of determination (R2) of 0.998, a mean absolute error (MAE) of 0.0.026, and a mean squared error (MSE) of 0.013. These metrics demonstrate the model’s exceptional capacity for generalization and precise identification of the underlying patterns in the data. The Physics-Informed Neural Network (PINN) model’s ability to predict power generation is evaluated using a scatter plot, as shown in Fig. 17. Most data points fall within the red dashed line, which denotes flawless prediction and shows how well the PINN model represents the underlying physical relationships. At mid-to-higher electricity values, however, there are minor underpredictions. With a physics-consistent learning framework, the model demonstrated strong predictive performance, characterized by a high coefficient of determination (R^2^) and Low statistical error on the validation set.

### Performance analysis of Stacking model, MATLAB Simulink-based physical model, and PINN model

**Fig. 18:**
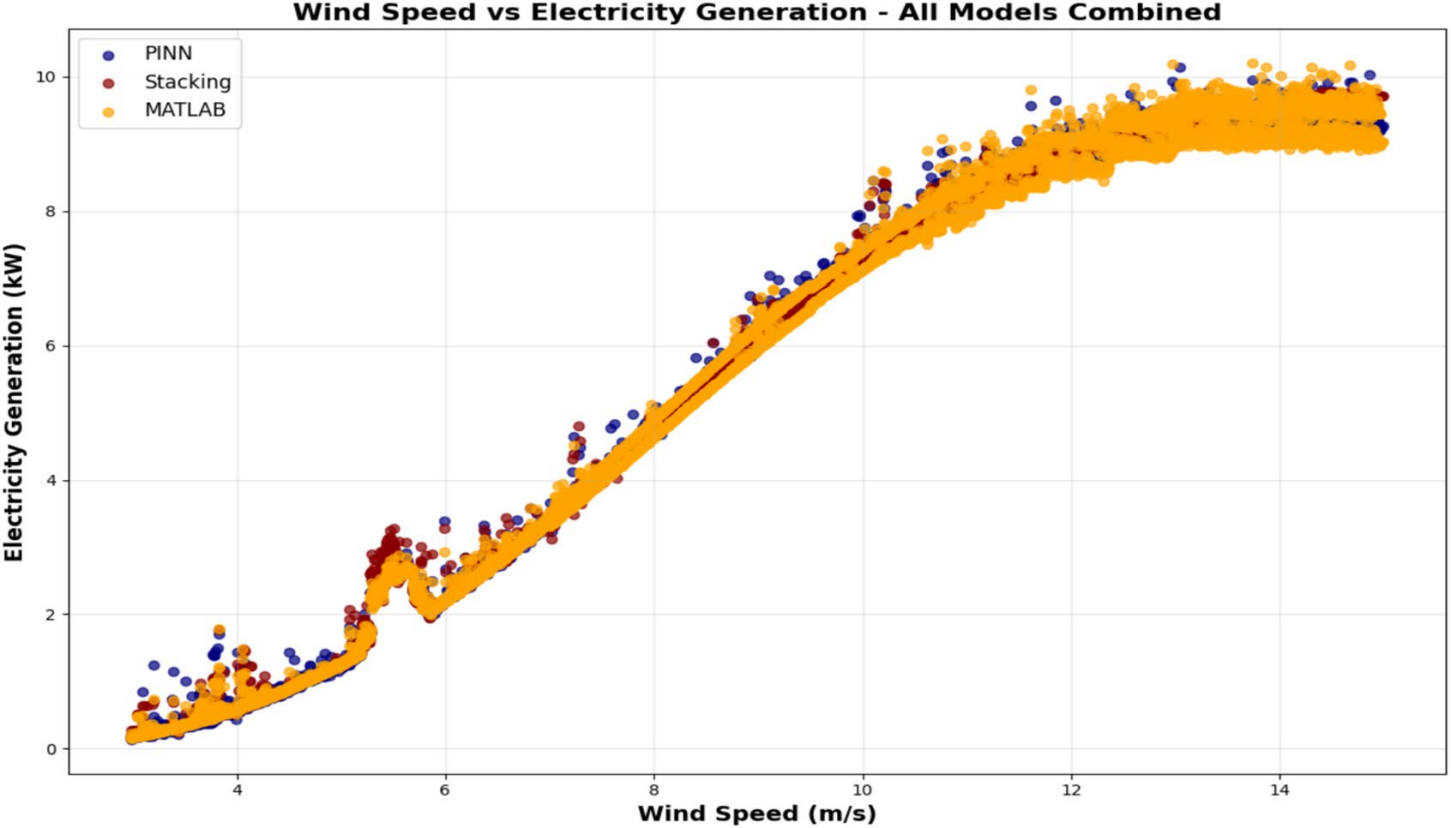
Comparison of the Physical model and the PINN model with ML-based Stacking models in a real-time dataset.

This section presents a comparative performance analysis of a machine learning (ML) model, a stacking ensemble, against a PINN model and a Physical model for wind energy generation, as illustrated in Fig. 18. Based on the combined scatter plot showing wind speed vs electricity generation for all three models, the analysis reveals comprehensive insights into their comparative performance. The plot illustrates the characteristic wind turbine power curve, comprising three operational phases: the cut-in region (3–6 m/s), the rated power region (6–12 m/s), following a cubic wind power relationship, and the rated capacity plateau (12–15 m/s), which stabilizes around 9.5–10 kW.

The Stacking ensemble model achieves the highest accuracy through a sophisticated combination of Random Forest and XGBoost. It demonstrates superior performance in complex transition regions (5–7 m/s), exhibiting the tightest clustering and excellent noise-handling capabilities. Its ensemble nature captures subtle patterns that individual algorithms might miss. The MATLAB physical model represents traditional engineering approaches based on established wind turbine theory. While exhibiting good performance, it shows more scatter in mid-range wind speeds, where real-world complexities deviate from idealized models. Overall, advanced machine learning approaches provide superior accuracy, with PINN offering an optimal balance between physical consistency and predictive performance. The PINN model exhibits exceptional adherence to wind power physics, demonstrating smooth transitions and effectively capturing the cubic relationship through its physics-informed architecture. It successfully incorporates knowledge of fluid dynamics and turbine aerodynamics, resulting in predictions that respect physical constraints with minimal scatter around the theoretical power curve.

#### Evaluation of Forecast Accuracy across Wind Speed ranges using Physical model, PINN model, and ML Models.

This section evaluates the forecasting accuracy of the Physical model, PINN model, and machine learning (ML) models across varying wind speeds. The Physical model exhibited high accuracy at wind speeds below the base value of 9 m/s, with a minimal error of 0.9%. However, its accuracy declined at higher wind speeds, with the error increasing to 2.9% beyond the rated speed of 11 m/s. This drop in precision is attributed to the complex aerodynamic and electromechanical behavior of wind turbines under high wind conditions, as well as the Physical models’ limited capacity to model rapid fluctuations in wind speed. In contrast, the ML model, namely, the Stacking Ensemble and PINN model, demonstrated strong accuracy and adaptability across the full spectrum of wind speeds. Their ability to capture the non-linear dynamics of wind energy generation resulted in more consistent and reliable predictions. Furthermore, ML models offered a significant reduction in computational time, making them more suitable for real-time applications and large-scale, long-term forecasting.

**Fig. 19:**
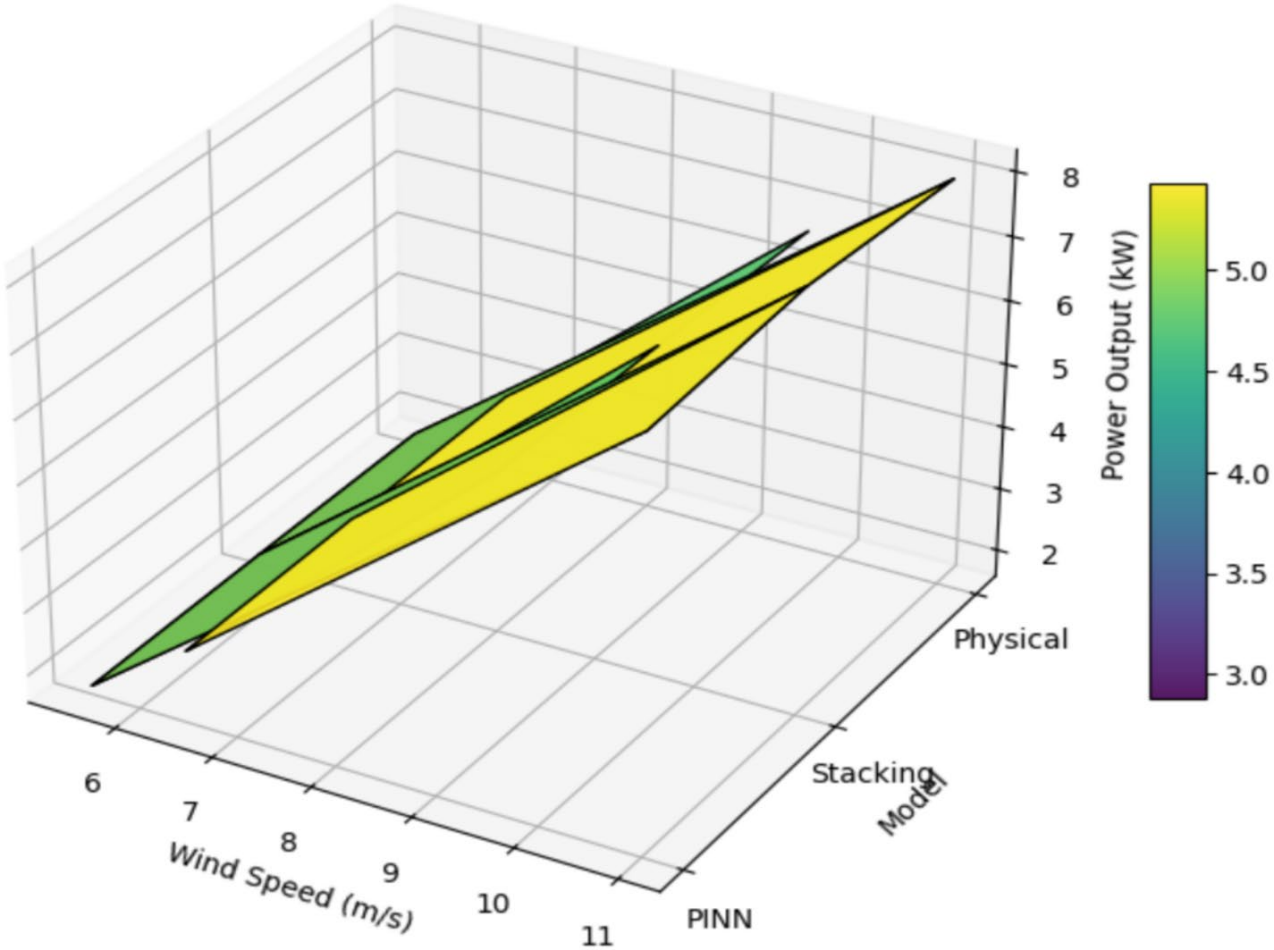
Comparison of Absolute Percentage Error Across Different Algorithms and Wind Speeds.

The forecasting results from the Stacking ensemble, PINN, and the Physical model were benchmarked against actual power output data obtained from the MERRA-2 dataset, as shown in Table 8. Five wind speed scenarios, ranging from 5.5 m/s to 11 m/s, were analyzed to evaluate the power generation estimates in kilowatts (kW). While each model demonstrated unique strengths at different wind speeds, the comparison highlighted only minor differences in forecast accuracy, underscoring the potential of ML models as robust alternatives to traditional simulation-based methods. The power output projections from three distinct models—PINN, Stacking Ensemble (RF + XGB), and the Physical Model—are visually compared across a range of wind speeds, from 5.5 m/s to 11 m/s, in the 3D surface graphic shown in Fig. 19. The X-axis represents Wind speed (m/s), the Y-axis encodes the model type (labelled PINN, Stacking, and Physical), and the Z-axis shows the related power output (kW). Across all models, the smooth, sloping surface shows a constant positive relationship between wind speed and expected power output. Notably, all three models exhibit close convergence at higher wind speeds (over 9 m/s), indicating outputs that are accurate and physically consistent. Minor variations are observed across the models at lower wind speeds, particularly between the Physical Model and the PINN, indicating sensitivity in the way each model responds to the nonlinear dynamics of wind energy conversion. These differences are further highlighted by the surface’s color gradient, which changes from purple (lower power) to yellow (greater power). The visualization shows that although all models show the anticipated trend of power growing with wind speed, Stacking and PINN behave more smoothly and broadly, indicating their significant learning capacity. This surface plot illustrates the effectiveness of data-driven and physics-based approaches in approximating complex interactions in wind power generation.

**Table 8:** Comparative Analysis of Wind Power Generation: Machine Learning Models vs MATLAB Simulation vs PINN at Various Sample Wind Speeds.

Sl. No	Wind speed (m/s)	Electricity generation (kW)			
		**ML Models**		**Physical Model (10 kW)**	**Actual value from the real-time data (MERRA-2) in kW**
		**PINN (kW)**	**Stacking (kW)**		
1	9.6	6.80	6.76	6.664	6.75
2	6.5	2.72	2.75	2.772	2.79
3	11	8.0	8.2	7.997	8.2
4	5.5	1.71	1.73	1.73	1.75
5	7.5	4.01	4.07	3.98	4.09

#### Uncertainty and computational analysis of all models

The evaluation of predictive models requires a comprehensive assessment that extends beyond traditional accuracy metrics to include uncertainty quantification and computational efficiency analysis, which are critical for real-world deployment in wind power forecasting systems. Uncertainty analysis provides essential insights into the reliability of predictions and confidence intervals, enabling informed decision-making and risk assessment in operational environments. Computational analysis, on the other hand, evaluates the practical feasibility of model deployment by examining factors such as training time, inference speed, memory requirements, and scalability constraints. This dual assessment approach is particularly vital for wind power prediction applications that demand real-time performance with high accuracy standards, ensuring that the selected models can operate effectively within the computational and temporal constraints of modern wind energy management systems.

**Fig. 20:**
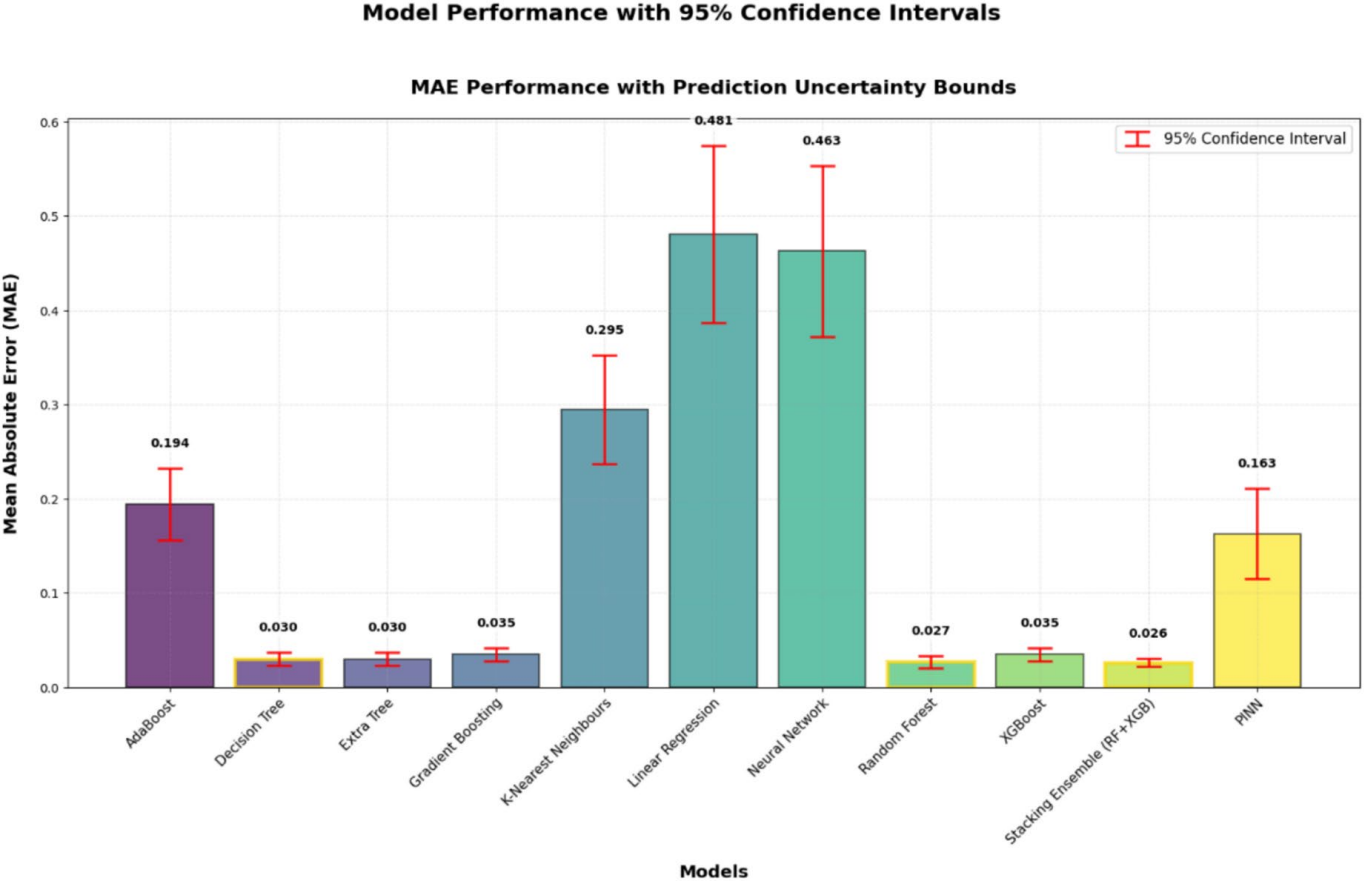
Comparative Model Performance with Prediction Uncertainty in Wind Energy Forecasting.

Figure 20 highlights the prediction uncertainty for each machine learning model using a bar chart, which displays the Mean Absolute Error (MAE) performance of the models along with their 95% confidence intervals. With the lowest MAE of 0.026 of all the models tested, the Stacking Ensemble (Random Forest + XGBoost) shows the best prediction accuracy and consistency. Random Forest (0.027) and Decision Tree/Extra Tree (0.030 each) come in close second and third, respectively. They all show low error and narrow confidence intervals, indicating good reliability. On the other hand, conventional models, such as Neural Network (MAE = 0.463) and Linear Regression (MAE = 0.481), exhibit much larger confidence bounds and higher error rates, indicating less accurate and uncertain predictions. When compared to ensemble and tree-based approaches, the K-Nearest Neighbours (0.295) and AdaBoost (0.194) models perform mediocrely but still fall short. With a somewhat higher MAE of 0.163, the Physics-Informed Neural Network (PINN) model outperforms traditional ensemble techniques, even surpassing simpler learners, such as Linear Regression. Even though PINNs might not outperform ensemble ML models in raw MAE, their unique strength lies in fusing scientific knowledge with learning ability, making them ideal for real-world applications where accuracy, robustness, and physical validity must coexist. As a result, PINNs are particularly useful in climates with physics constraints, such as wind energy systems, and in forecasts enhanced by simulation.

**Fig. 21:**
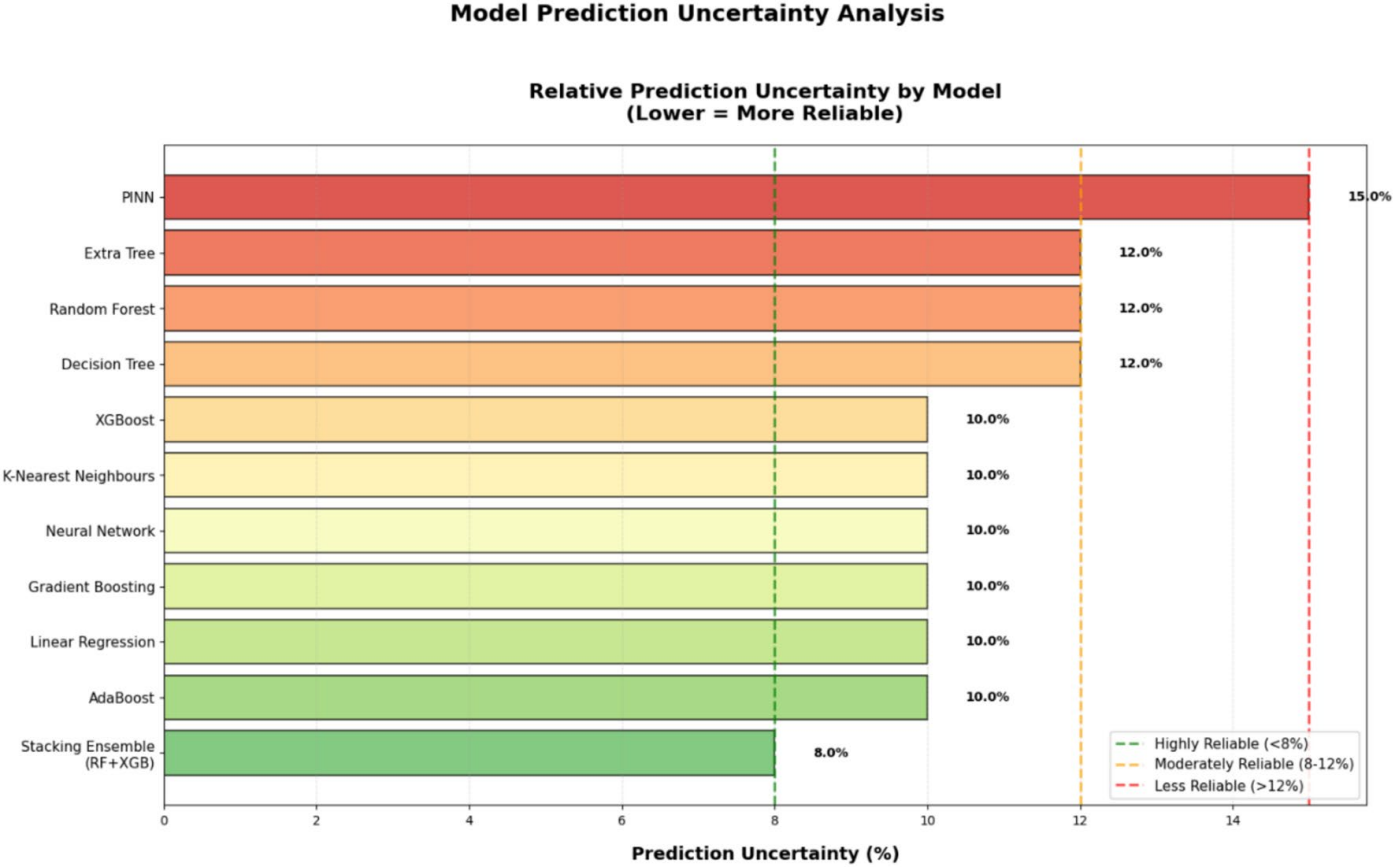
Model Prediction Uncertainty Analysis in Renewable Energy Forecasting.

**Fig. 22:**
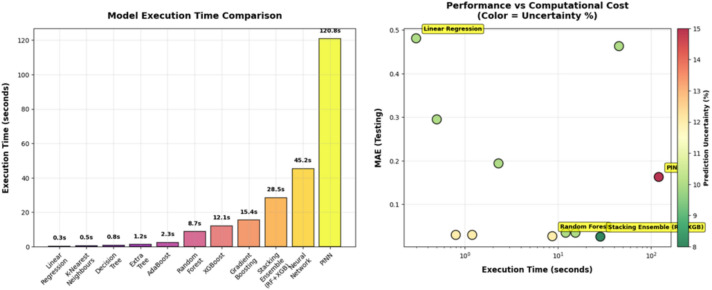
Comparative Analysis of Model Execution Time, Accuracy, and Prediction Uncertainty for Renewable Energy Forecasting.

Figure 21 compares the prediction uncertainty of several machine learning models; a smaller uncertainty denotes a more reliable model. The Stacking Ensemble (Random Forest + XGBoost) falls into the Highly Reliable category with the lowest prediction uncertainty of 8%. Most other models, such as AdaBoost, K-Nearest Neighbours, Linear Regression, Gradient Boosting, and Neural Network, are in the 10% range for Moderately Reliable. The uncertainty of models such as Extra Tree, Random Forest, and Decision Tree is marginally higher at 12%. The Physics-Informed Neural Network (PINN) model is classified as Less Reliable because it exhibits the highest level of uncertainty, at 15%. However, this does not make PINNs any less valuable. PINNs provide special benefits by incorporating physical system knowledge (such as differential equations and energy conservation principles) straight into the learning process, even if their raw prediction output is more variable. Therefore, even though PINNs might not be the most dependable when considering statistical uncertainty alone, they offer substantial long-term advantages in terms of credibility, model resilience, and practicality in challenging, physics-restricted fields. In Fig. 22, models are compared according to prediction uncertainty, MAE, and execution time. While XGBoost (15.4 s), Random Forest (12.1 s), and Stacking Ensemble (28.5 s) give low MAE with minimum uncertainty, making them efficient and dependable, Linear Regression is the fastest (0.3 s) but least accurate. PINN is better suited for physics-informed scenarios that demand deeper interpretability, as it achieves good accuracy but has the maximum execution time (120.8 s) and larger uncertainty. A detailed analysis and comparison with existing literature were conducted for the best-performing ML model and the PINN model.

### Case 4: Hybrid Forecasting for 2026 – Integrating Machine Learning with MATLAB Simulink for Wind Power Prediction in Aralvaimozhi

In this case study, a hybrid forecasting approach was employed by integrating a machine learning (ML) model with a Physical model and PINN to predict wind power generation for the year 2026 in the Aralvaimozhi region. Historical wind speed data from the MERRA-2 open-access platform, as used in Cases 1, 2, and 3, served as the foundation for forecasting future wind conditions.

**Fig. 23:**
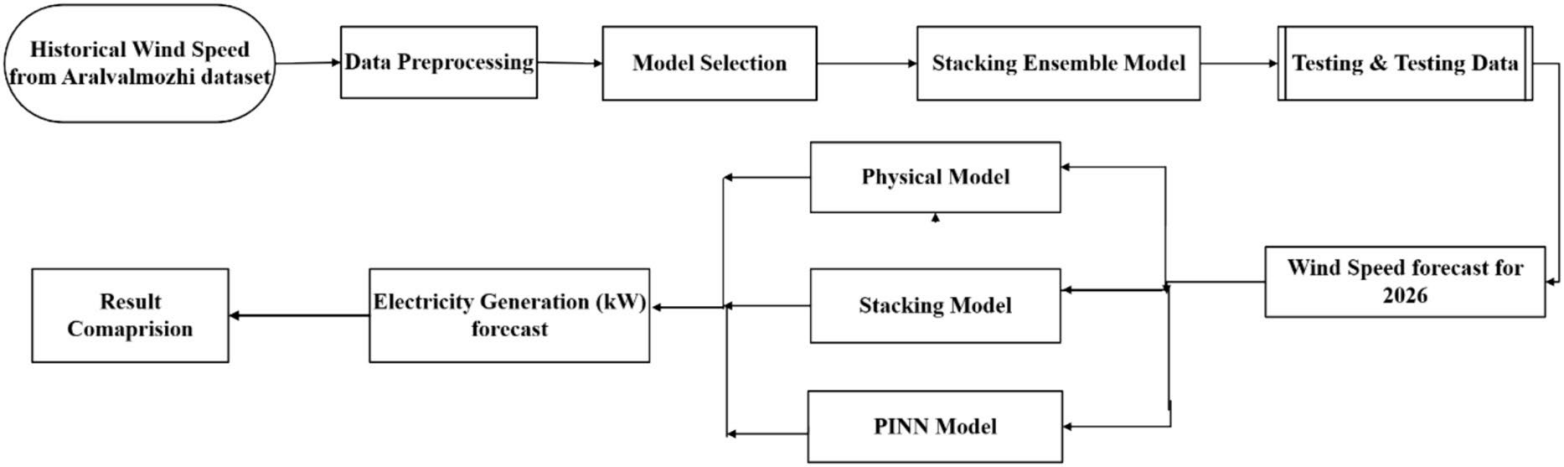
Workflow for Wind Speed Forecasting and Electricity Generation Prediction Using Stacking Ensemble Model with Physical Model and PINN Model (2026).

The predictions accounted for an annual decline in average wind speed of up to 0.6%, as reported by the Council on Energy, Environment, and Water (CEEW), New Delhi. This decline factor was incorporated based on key parameters, including wind speed range (3–15 m/s) and temporal variability. A stacking ensemble model, combining Random Forest and XGBoost algorithms, was used to generate wind speed forecasts. These predicted values were then input into a Physical model to simulate the corresponding power output from a 10-kW wind energy conversion system. A total of 8,670 hourly data points for 2026 were evaluated. The PINN utilizes prediction from the Stacking model, and values for physics variables from the Physical model are visualized in Fig. 23.

**Fig. 24:**
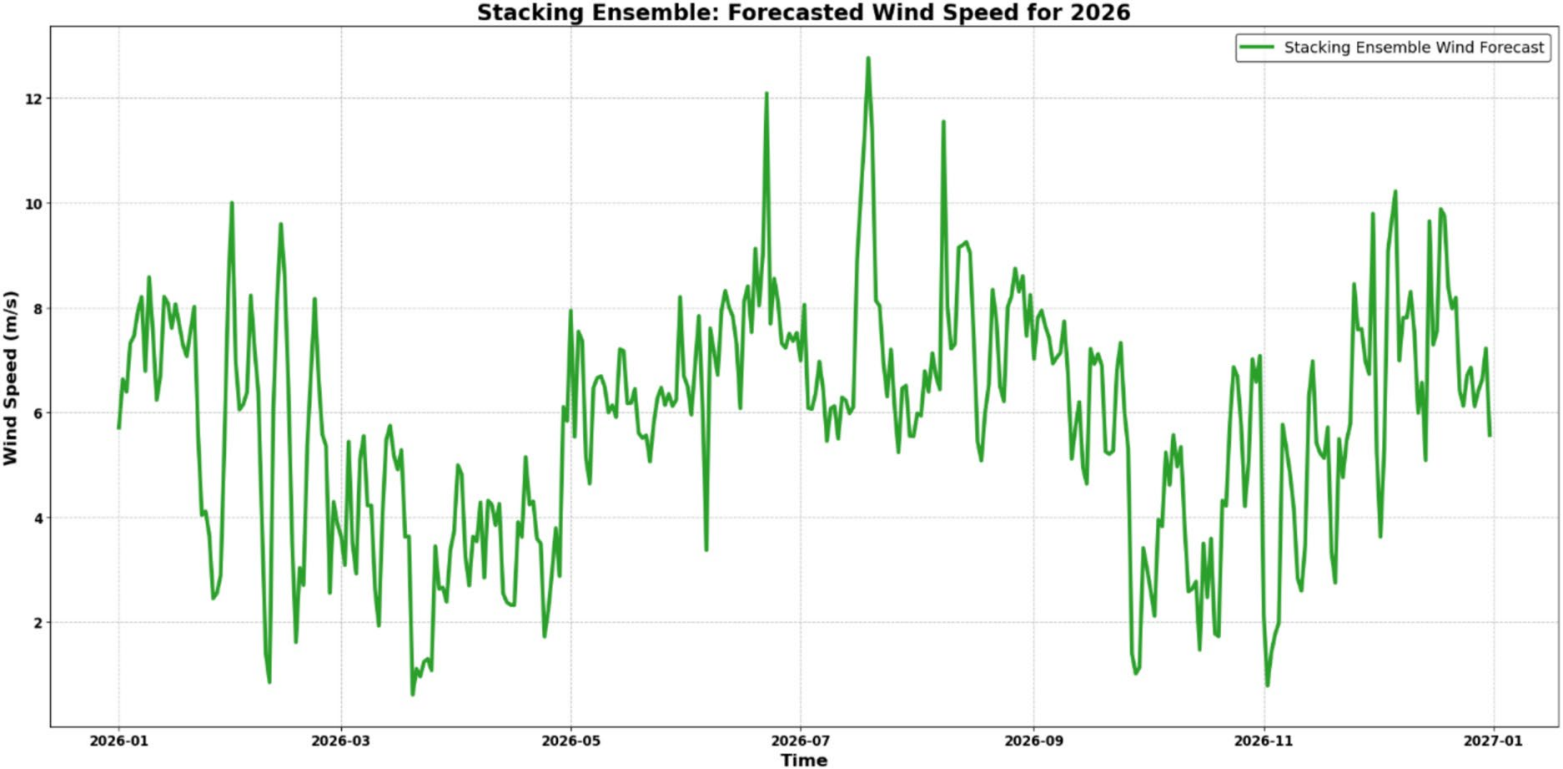
Stacking Ensemble: Forecasted Wind Speed for 2026.

Figure 24 illustrates the forecasted hourly wind speed for 2026, as predicted by the Stacking Ensemble model, based on historical data trends and model predictions. The X-axis represents the period spanning from January 2026 to January 2027, while the Y-axis shows the predicted wind speed in meters per second (m/s). The plot reveals noticeable seasonal and temporal variations in wind patterns, with wind speeds peaking during the mid-year months (June to August), reaching values above 12 m/s, likely corresponding to the monsoon period in southern India. In contrast, lower wind speeds are observed during March to May and parts of October, with values frequently dipping below 4 m/s, indicating relatively calm conditions. The forecast also captures the intermittent and fluctuating nature of wind energy, which is crucial for planning and managing wind-based power generation. The Stacking model’s output reflects its robustness in capturing non-linear patterns and variability over time, making it a reliable tool for long-term wind resource forecasting. This forecast data serves as a key input for subsequent modeling of electricity generation using both machine learning and physics-based approaches.

Table 9 presents a summary of five representative forecast instances, comparing the power output predictions from the stacking ensemble model with those obtained from the Physical model and the PINN model. For instance, in Table 9, instance 2, a high wind speed of 14.408 m/s resulted in predicted power outputs of 9.72 kW from the stacking model, 9.45 kW from the Physical model, and 9.67 kW from PINN. Conversely, in Instance 5, a lower wind speed of 4.872 m/s produced outputs of 1.17 kW, 1.15 kW, and 1.16 kW, respectively. Across all instances, the stacking model consistently predicted slightly higher power generation, suggesting a greater sensitivity or a modest optimistic bias when compared to Physics-based Models.

**Table 9:** Comparison of Forecasted Wind Speed and Electricity Generation Using Stacking Ensemble Model and MATLAB Simulation (10 kW) for 2026.

Sl. No	Forecasted Wind speed (m/s)	Forecasted Electricity generation (kW)		
		**Stacking Ensemble Model**	**Physical Model (10 kW)**	**PINN model**
1	7.494	4.06	3.96	4.01
2	14.408	9.72	9.45	9.67
3	11.783	8.66	8.5	8.62
4	10.184	7.46	7.3	7.41
5	4.872	1.17	1.15	1.16

**Fig. 25:**
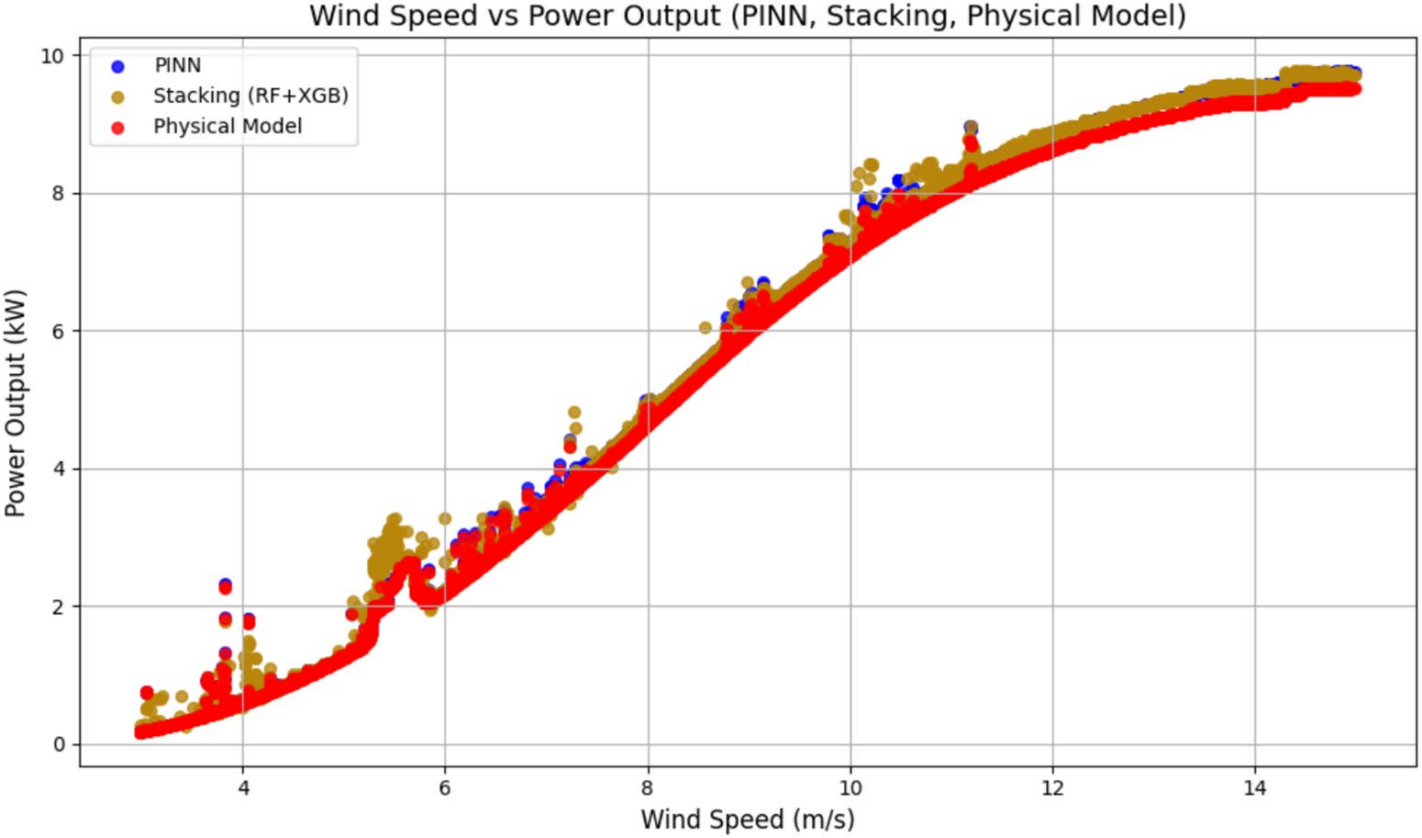
Wind Speed vs. Stacking (RF + XGB), MATLAB/Simulink, and PINN Power Outputs.

Figure 25 presents the power-wind speed relationship as predicted by the three models. All models follow a typical non-linear wind turbine power curve, starting near 0 kW at wind speeds below 3 m/s, rising sharply between 4 and 10 m/s, and plateauing at around 10 kW beyond 12 m/s. While the overall trends align, the stacking ensemble model predicts marginally higher outputs, particularly at intermediate wind speeds (6–10 m/s). This suggests the ML model’s enhanced responsiveness to subtle wind speed variations, while both models converge at the lower and upper bounds. The integration of data-driven ML forecasting with physical modeling provides a robust and comprehensive toolset for wind energy planning. This hybrid approach enables more accurate, timely, and adaptable forecasting, supporting effective decision-making for renewable energy deployment in 2026.

## Conclusion

This study presents a comprehensive hybrid forecasting framework that integrates machine learning models, MATLAB Simulink-based physical modeling, and Physics-Informed Neural Networks to enhance the accuracy, scalability, and interpretability of wind power prediction. Through systematic evaluation of multiple ML algorithms, the Stacking ensemble model combining Random Forest and XGBoost achieved superior performance with R^2^ = 0.998 and RMSE = 0.11, demonstrating consistent high accuracy across varying wind speeds while maintaining low computational demand suitable for real-time and large-scale forecasting applications. While the MATLAB Simulink-based physical model proved effective at lower wind speeds, its accuracy diminished at higher wind velocities due to increased computational complexity and limitations in representing nonlinear aerodynamic dynamics. To address these challenges, a Physics-Informed Neural Network was developed that incorporated both observational and synthetic physics-based data, ensuring physically consistent forecasts and providing improved generalization in scenarios with limited or noisy data. The hybrid framework’s practical applicability was validated through a forward-looking case study forecasting wind power generation for 2026 in southern Tamil Nadu, where the Stacking ensemble model’s forecasts were cross verified using the Simulink and PINN models, demonstrating strong alignment across all approaches. This work highlights the significant value of combining data-driven and physics-based techniques for robust, accurate, and interpretable wind power forecasting, thereby supporting informed planning and real-time decision-making, and offering clear benefits for grid reliability, operational efficiency, and sustainability. The framework aligns with Sustainable Development Goals, particularly SDG 7 (Affordable and Clean Energy) and SDG 13 (Climate Action), contributing to global efforts toward renewable energy transition. Future research opportunities include validating the framework across diverse geographic regions and turbine technologies to assess its generalizability. Additionally, incorporating advanced deep learning architectures, such as Long Short-Term Memory networks, Transformer-based models, and ensemble deep networks, can improve temporal resolution and accuracy for larger datasets. Real-time integration of meteorological and SCADA data streams will enable adaptive retraining, ensuring models remain responsive to changing conditions. Incorporating probabilistic forecasting methods will enhance uncertainty quantification for risk-aware decision-making. Ultimately, deploying the framework on edge or cloud platforms and exploring digital twin architectures can support real-time optimization, predictive maintenance, and intelligent control in innovative grid environments, advancing the field toward more sophisticated and reliable wind power forecasting systems.

## Data Availability

Renewables.ninja Wind Data (MEERA-2 Dataset): Wind resource data used in this study was sourced from Renewables.ninja, an open-access platform for renewable energy modeling data. The dataset is accessible at: https://www.renewables.ninja Wind Power Generation Data – Forecasting: Data used in this study was sourced from Kaggle licenced under CC0 public domain. The dataset is accessible at: https://www.kaggle.com/datasets/mubashirrahim/wind-power-generation-data-forecasting.
